# TURBOMOLE: Modular program suite for *ab initio* quantum-chemical and condensed-matter simulations

**DOI:** 10.1063/5.0004635

**Published:** 2020-05-13

**Authors:** Sree Ganesh Balasubramani, Guo P. Chen, Sonia Coriani, Michael Diedenhofen, Marius S. Frank, Yannick J. Franzke, Filipp Furche, Robin Grotjahn, Michael E. Harding, Christof Hättig, Arnim Hellweg, Benjamin Helmich-Paris, Christof Holzer, Uwe Huniar, Martin Kaupp, Alireza Marefat Khah, Sarah Karbalaei Khani, Thomas Müller, Fabian Mack, Brian D. Nguyen, Shane M. Parker, Eva Perlt, Dmitrij Rappoport, Kevin Reiter, Saswata Roy, Matthias Rückert, Gunnar Schmitz, Marek Sierka, Enrico Tapavicza, David P. Tew, Christoph van Wüllen, Vamsee K. Voora, Florian Weigend, Artur Wodyński, Jason M. Yu

**Affiliations:** 1 Department of Chemistry, University of California, Irvine, 1102 Natural Sciences II, Irvine, California 92697-2025, USA; 2 DTU Chemistry, Technical University of Denmark, Kemitorvet Build. 207, DK-2800 Kongens Lyngby, Denmark; 3 Dassault Systèmes Deutschland GmbH, Imbacher Weg 46, 51379 Leverkusen, Germany; 4 Lehrstuhl für Theoretische Chemie, Ruhr-Universität Bochum, 44801 Bochum, Germany; 5 Institute of Physical Chemistry, Karlsruhe Institute of Technology (KIT), KIT Campus South, P.O. Box 6980, 76049 Karlsruhe, Germany; 6 Institut für Chemie, Theoretische Chemie/Quantenchemie, Technische Universität Berlin, Sekr. C7, Straße des 17. Juni 135, 10623 Berlin, Germany; 7 TURBOMOLE GmbH, Litzenhardtstraße 19, 76135 Karlsruhe, Germany; 8 Max-Planck-Institut für Kohlenforschung, Kaiser-Wilhelm-Platz 1, 45470 Mülheim an der Ruhr, Germany; 9 Forschungszentrum Jülich, Jülich Supercomputer Centre, Wilhelm-Jonen Straße, 52425 Jülich, Germany; 10 Department of Chemistry, Case Western Reserve University, 10900 Euclid Avenue, Cleveland, Ohio 44106, USA; 11 Department of Chemistry, University of North Carolina at Chapel Hill, Chapel Hill, North Carolina 27599, USA; 12 Institute of Nanotechnology, Karlsruhe Institute of Technology (KIT), KIT Campus North, P.O. Box 3640, 76021 Karlsruhe, Germany; 13 Department of Chemistry, Aarhus Universitet, Langelandsgade 140, DK-8000 Aarhus, Denmark; Otto-Schott-Institut für Materialforschung, Friedrich-Schiller-Universität Jena, Löbdergraben 32, 07743 Jena, Germany; 15 Department of Chemistry and Biochemistry, California State University, Long Beach, 1250 Bellflower Boulevard, Long Beach, California 90840, USA; 16 Max Planck Institute for Solid State Research, Heisenbergstaße 1, 70569 Stuttgart, Germany; 17 Physical and Theoretical Chemistry Laboratory, University of Oxford, South Parks Road, Oxford OX1 3QZ, United Kingdom; 18 Fachbereich Chemie and Forschungszentrum OPTIMAS, Technische Universität Kaiserslautern, Erwin-Schrödinger-Staße 52, 67663 Kaiserslautern, Germany; 19 Department of Chemical Sciences, Tata Institute of Fundamental Research, Homi Bhabha Road, Colaba, Mumbai 400005, India; 20 Fachbereich Chemie, Philipps-Universität Marburg, Hans-Meerwein-Str. 4, 35032 Marburg, Germany

## Abstract

TURBOMOLE is a collaborative, multi-national software development project aiming to provide highly efficient and stable computational tools for quantum chemical simulations of molecules, clusters, periodic systems, and solutions. The TURBOMOLE software suite is optimized for widely available, inexpensive, and resource-efficient hardware such as multi-core workstations and small computer clusters. TURBOMOLE specializes in electronic structure methods with outstanding accuracy–cost ratio, such as density functional theory including local hybrids and the random phase approximation (RPA), *GW*-Bethe–Salpeter methods, second-order Møller–Plesset theory, and explicitly correlated coupled-cluster methods. TURBOMOLE is based on Gaussian basis sets and has been pivotal for the development of many fast and low-scaling algorithms in the past three decades, such as integral-direct methods, fast multipole methods, the resolution-of-the-identity approximation, imaginary frequency integration, Laplace transform, and pair natural orbital methods. This review focuses on recent additions to TURBOMOLE’s functionality, including excited-state methods, RPA and Green’s function methods, relativistic approaches, high-order molecular properties, solvation effects, and periodic systems. A variety of illustrative applications along with accuracy and timing data are discussed. Moreover, available interfaces to users as well as other software are summarized. TURBOMOLE’s current licensing, distribution, and support model are discussed, and an overview of TURBOMOLE’s development workflow is provided. Challenges such as communication and outreach, software infrastructure, and funding are highlighted.

## I. INTRODUCTION

The aim of the TURBOMOLE project is to provide highly efficient and stable computational tools for quantum chemical calculations on presently affordable and widely available hardware, such as multi-core workstations and small computer clusters. TURBOMOLE focuses on electronic structure methods such as density functional theory (DFT), second-order Møller–Plesset (MP2) theory, random-phase approximation (RPA) methods, and coupled-cluster (CC) theory. TURBOMOLE’s integral processing was developed and optimized for segmented-contracted Gaussian basis sets, frequently developed concomitantly with the code. Typical TURBOMOLE applications involve structure optimizations and transition-state searches in ground and electronically excited states, calculations of energies and thermodynamic functions as well as optical, electric, and magnetic properties, and *ab initio* molecular dynamics simulations within and beyond the Born–Oppenheimer (BO) approximation. For condensed matter simulations, an efficient implementation of periodic boundary conditions, solvation models such as the conductor-like screening model (COSMO), and more general atomistic electrostatic and polarizable embeddings are available.

TURBOMOLE currently has over 50 active developers on three continents and 500–1000 active end-user licenses corresponding to thousands of users in academia, education, government, and industry world-wide. A Google Scholar search for “TURBOMOLE” yields ∼1070 hits for 2018 and 16 000 for all years.^1^

TURBOMOLE’s efficiency derives from its focus on methods with outstanding cost-to-performance ratios, implemented using extensively optimized algorithms that combine speed with low-order scaling with system size: Low-order scaling is primarily achieved by integral direct algorithms,^2–6^ exploiting sparsity^3,7–14^ and point group symmetry.^15–17^ Resolution-of-the-identity (RI) methods,^9,10,13,18–33^ numerical Laplace transform,^12,34^ and shared and distributed memory parallelizations^26,35–42^ provide substantial additional acceleration.

This review is written primarily for readers interested in using electronic structure software for specific applications. Thus, after an overview of its design philosophy (Sec. II), the main body of this review focuses on examples and applications that illustrate and provide context for recent methodological additions to the program suite (Sec. III). Section III demonstrates the capability of TURBOMOLE by applications to thermochemistry, ground-state potential energy surfaces (Secs. III A–III F), spectroscopic characterization (Secs. III G–III R), and embedding and solvation (Secs. III S–III V). Besides potential and current TURBOMOLE users, this review also addresses readers interested in computational method development looking to interface their own code with TURBOMOLE or participate in the development. Section IV describes the existing interfaces of TURBOMOLE and the surrounding software ecosystem, while Sec. V summarizes the current development and licensing model. Some current challenges are discussed in Sec. VII. Detailed information on recent releases, how to obtain a TURBOMOLE license, and a comprehensive manual of TURBOMOLE’s functionality are available on the TURBOMOLE website.^43^ A brief history of the project is provided in the supplementary material.

## II. TURBOMOLE DESIGN PRINCIPLES

### A. Modular structure

The TURBOMOLE program suite consists of almost 25 main executables and more than 100 auxiliary programs, interactive tools, and scripts (referred to as modules further on), as well as separate basis set and structure libraries, documentation, and an automated test suite. The modular structure enables encapsulation of functionality. For example, input generation, Hartree–Fock (HF) or Kohn–Sham (KS) self-consistent field (SCF) energy calculations, nuclear gradients, and structure optimization are implemented in separate executables. Higher-level workflows such as equilibrium^44^ or transition state structure searches^45,46^ and reaction pathway optimization^47^ are implemented through scripts.

TURBOMOLE uses simple metadata descriptors called “data groups” to organize input/output (I/O) operations. All metadata are gathered in the “control” file, which is the only required input file for TURBOMOLE calculations. The control file is human-readable plain text American standard code for information interchange (ASCII) and may contain external file references for data groups such as coordinates, energies, gradients, or molecular orbital (MO) coefficients. Each TURBOMOLE module reads and possibly updates the data provided by the control file.

The modular structure of TURBOMOLE gives users flexibility to implement their own workflows. Moreover, it allows users to track progress and perform manually each step of a calculation if they choose to, which is particularly helpful for educational purposes. The command-line interface and simple, human-readable I/O helps to achieve UNIX-like robustness and extensive, automated applications. However, the flexibility of the command-line tools and the multitude of options can be daunting. Thus, new users often find it easier to start with a graphical interface (see Sec. IV B).

### B. Available methods

While there are almost unlimited possibilities to combine TURBOMOLE’s building blocks into larger workflows, the methods and algorithms implemented in TURBOMOLE represent a carefully selected choice based on the following criteria:

• broad applicability and transferability,
• outstanding accuracy and predictive power per kWh of energy consumed,
• stability and demonstrated performance,
• scientific soundness and limited empiricism,
• transparency and reproducibility across applications, operating systems, and versions, and
• focus on observable quantities.

Additional considerations include requests from users and scientific interests and expertise of the developers. The decision to support or not support certain methods or functionalities can be contentious and requires careful weighing of the above criteria, requests of different groups of users and developers, and costs for maintenance and support. TURBOMOLE GmbH uses a proposal-based process to evaluate requests for new methods and features; the final decision is made by vote of the TURBOMOLE stakeholders (see Sec. V).

The current scope of the TURBOMOLE project does not extend to approaches such as highly parameterized semi-empirical methods, high-end correlation treatments for spectroscopic accuracy, most multi-reference methods, or model Hamiltonians. Users and developers interested in such approaches are referred to other papers in this special topic.

### C. Self-consistent field methods

A central part of most quantum chemistry codes are Hartree–Fock and DFT SCF programs. In modern semi-direct SCF implementations, construction of the Fock or KS matrix and the evaluation and processing of electron repulsion integrals (ERIs) dominate floating point and memory operation count in the vast majority of applications, especially in conjunction with parallel linear algebra and diagonalization algorithms (see Sec. II I). TURBOMOLE uses hand-optimized subroutines^48^ for low angular momentum ERIs and the Obara–Saika algorithm for higher angular momenta. Specially adapted algorithms^49^ are used for three- and two-index integrals necessary for the RI approximation to the Coulomb (RI-*J*)^19,20^ and exchange integrals (RI-*K*).^23^

Integral prescreening along with the difference density method is used to achieve quadratic scaling of the timings for Fock matrix builds with the system size *N* for large molecules.^3,50^ Furthermore, the direct inversion of the iterative subspace (DIIS) method^51^ is available to reduce the number of SCF iterations. The multipole-accelerated RI-*J* (MARI-*J*) approach^27^ in the ridft module and a combination of RI and a continuous fast multipole method (CFMM)^9^ in the riper module can be used to further speed up calculations on large molecules and periodic systems, respectively, and achieve near linear scaling for the Fock matrix builds. All four semi-local rungs of Jacob’s ladder^52^ of exchange–correlation (XC) functionals are supported, including local spin density approximation (LSDA) and generalized gradient approximation (GGA) functionals, meta-GGAs, as well as global, range-separated, and local hybrid functionals. Moreover, energies can be obtained with the double hybrid functional B2-PLYP.^53^ Interfaces to the LIBXC^54^ and XCFUN^55^ libraries are provided to support a wide range of functionals. Molecular grids are constructed by Becke partitioning^56^ of the optimized atomic grids based on radial Gauss–Chebyshev integration and spherical Lebedev integration.^57^ For periodic systems, a linear scaling hierarchical integration scheme is available.^58^ All semi-local XC integration schemes exploit the locality of Gaussian basis functions by sorting grid points into relatively compact “batches,” enabling strictly linear scaling of the XC quadrature for energies, XC potentials, and derivative properties.^59^

In all HF and DFT modules, general petite-list algorithms^15,16^ for the calculation and processing of molecular integrals and a reduction of the numerical quadrature points to symmetry-unique ones^60^ are used to exploit symmetry for Abelian and non-Abelian point groups. This leads to a speed-up by the order of the point group and reduces the memory demands as only a skeleton quantity has to be stored. Moreover, the Clebsch–Gordan reduction of tensor operators in the molecular orbital basis is employed in several parts of the code to speed up transformations and linear algebra steps.^61^

Since TURBOMOLE’s integral evaluation procedures are highly optimized for segmented-contracted Gaussian basis sets and low memory demands, they are not optimal for general-contracted basis sets, which are very frequently employed in post-HF, especially coupled-cluster, calculations. Still, application of the RI approximation with proper auxiliary basis sets and the usage of so-called “optimized general contractions”^62^ result in a significant improvement.^63^ However, these approaches could be seen as a simple “work-around” only.

### D. HF and DFT response theory and molecular properties

The solution of SCF equations to obtain the ground-state energy is often only the first step of a computational workflow. Ground-state properties are defined by the response of the ground-state energy or action to external perturbations. The reader is referred Ref. 64 for a recent review. According to Wigner’s (2*n* + 1) rule, the wavefunction response of up to *n*th order is sufficient to compute the response properties of order (2*n* + 1). As a result, second-order properties such as electronic polarizabilities,^65^ nuclear magnetic resonance (NMR) chemical shifts,^4^ and vibrational frequencies^66–68^ can all be obtained from solving linear response (LR) equations with respect to electric, magnetic, and nuclear displacement perturbations, respectively. Calculations of excitation energies and transition moments involve the solution of closely related eigenvalue equations and can be performed within the same implementation.^69^ The common algorithm employs iterative Davidson^5,33,61^ or non-orthonormal Krylov space procedures^10^ and typically converges in fewer iterations than the corresponding ground-state SCF calculation.^10^ The computationally intensive steps of the iterative procedure consist in the contraction of trial vectors with ERIs and the matrix elements of the exchange-correlation kernel. Importantly, these steps are akin to the Fock matrix construction in ground-state SCF calculations^5^ and thus can take advantage of techniques such as integral prescreening,^5^ recursive matrix build,^10^ and use of point group symmetry.^5^ However, the symmetry of the perturbation must be taken into account when computing response properties, for example, a homogeneous magnetic field transforms as an axial vector.^17^

Linear scaling of the operation count for all operations related to the response of the XC energy is achieved by recognizing that a single LR iteration is equivalent to the construction of a linearized XC potential. For meta-GGAs, the current density response is needed in addition to the response of the density, gradient, and kinetic energy density to ensure consistent response properties and gauge-invariance.^70^ Moreover, within the non-collinear DFT framework, spin-flip excitations can be obtained in time-dependent DFT (TDDFT).^71^

Photochemical studies depend on efficient implementations of excited-state energy derivatives.^61,72,73^ The Lagrangian formalism makes it possible to compute excited-state gradients at a constant multiple of the cost of ground-state gradients. This is achieved by re-formulating the expression for the excitation energy within time-dependent HF (TDHF) or TDDFT as a variational functional of all parameters, including molecular orbital coefficients.^24,74^ As a result, no MO coefficient derivatives with respect to nuclear displacements need to be computed, in analogy to the Hellmann–Feynman theorem for ground-state gradients. Instead, an additional linear equation needs to be solved to obtain the Lagrange multipliers. Given the excitation vectors and the Lagrange multipliers, the excited-state energy gradients are computed by contraction with integral derivatives. The Lagrangian approach is also applicable to electronic polarizabilities and allows for an efficient computation of Raman cross sections.^75^

The total computational cost of TDHF and TDDFT response calculations scales as $O\left(N^{2}\;n_{b}\right)$ with the basis size *N* and the number of batches *n*_*b*_ used in the block Davidson procedure.^61^ Calculations of electronic polarizabilities and NMR shifts treat a constant number of perturbations (one for each external field component) and can be performed with *n*_*b*_ = 1 batch if the trial vectors fit into main memory. Similarly, electronic excitation calculations usually include only few excited states and can be accommodated within a *n*_*b*_ = 1 batch. NMR shifts are still cheaper to compute due to a simpler structure of the matrix elements of the response matrix for symmetry reasons.^4,26^ The calculations of these properties are routinely feasible for molecular systems of 100–1000 atoms.^26,61,72,76^ In contrast, calculations of vibrational frequencies involve 3 *n*_*a*_ geometric perturbations, where *n*_*a*_ is the number of atoms. While batching is still helpful in boosting efficiency, vibrational frequency calculations are subject to $O\left(N^{2}\;n_{a}\right)$ scaling.^66–68^

### E. Correlated wavefunction methods

Until the mid-1990s, MP2 and related second-order methods using triple-*ζ* or larger basis sets were limited to molecules with no more than ∼10 symmetry-distinct non-hydrogen atoms. The main limitation was the large prefactor of the atomic orbital (AO) to MO transformation of the four-index electron repulsion integrals and the associated storage and I/O demands.^6,77,78^ For the approximate coupled-cluster singles and doubles method (CC2) and algebraic-diagrammatic construction through second-order [ADC(2)], the storage demands for doubles amplitudes was another obstacle for large-scale calculations. This problem was solved when Weigend *et al.* combined the RI approximation with the optimized auxiliary basis sets for MP2 calculations.^30,79^ The underlying factorization of two-electron integrals was the basis for a highly efficient implementation for a wide range of correlated wavefunction methods, including the iterative second-order response methods ADC(2) and CC2 in the ricc2 module.^22,38,80,81^ The RI factorization fully avoids AO to MO transformations of four-index integrals and the storage and I/O of any four-index intermediates. To facilitate large-scale calculations on standard hardware, only quantities that scale at most as $O\left(N^{3}\right)$ with the basis size *N* and linearly with the number of states and perturbations are stored on disk. Batching is used to exploit available random access memory (RAM) to reduce I/O while ensuring that the minimum demands for RAM increase only as $O\left(N^{2}\right)$ with the basis set.^22,38^ The implementation was later extended to the spin-component scaled (SCS) and scaled opposite-spin (SOS) variants of ADC(2) and CC2.^34,82,83^

With release V6.2, the software suite was extended by an explicitly correlated^84^ canonical CCSD and CCSD(T) code. At this level, the storage of doubles amplitudes is unavoidable. The code, now available in the ccsdf12 module, uses integral-direct and RI techniques to avoid four-index transformations and storage of quantities with more than two virtual indices. For CCSD(T), in addition a file with the four-index integrals with three virtual indices and one occupied index is precalculated and stored on disk. Batching algorithms are used to arrive at minimum RAM demands that increase at most as $O\left(nN^{2}\right)$ with the basis set size and the number of electrons *n*. In contrast to most other CCSD codes, the implementation in TURBOMOLE uses, in the time-determining steps, outermost loops running over virtual and innermost loops running over occupied orbitals. While less efficient for tiny molecules with huge basis sets, this structure leads to a much better thread-parallelization for larger molecules and the typical medium-sized basis sets that are used in explicitly correlated CCSD(F12^*^) calculations.^84,85^

The steep increase in the operation count of $O\left(N^{6}\right)$ and $O\left(N^{7}\right)$ with the system size limits the applicability of canonical CCSD and CCSD(T) still to rather small systems (cf. Secs. III A and III L). This is attenuated in the pnoccsd program that uses pair natural orbitals (PNOs) for the virtual and localized orbitals for the occupied space. The local approximations allow to screen out negligible contributions and thereby reduce the scaling of the computational costs with the system size until ultimately almost linear scaling is achieved. Similar to other PNO-based programs, the implementation additionally exploits projected atomic orbitals (PAOs) and orbital-specific virtuals (OSVs) as intermediate basis, local RI approximations, and a hierarchy of pair approximations. By default, all screening thresholds are calculated from the PNO threshold such that it is the latter approximation that determines the deviation from the canonical result.^14,86,87^

### F. Periodic systems

For nearly 30 years, the functionality of TURBOMOLE was limited to molecules. In 2015, with release V7.0, it was extended to DFT calculations applying periodic boundary conditions, which enables calculations on periodic systems such as chains, polymers, surfaces, or crystals.^9^ To facilitate an efficient implementation, the new riper program was designed and written from scratch, reusing only the most efficient integral subroutines of the existing code. Several new algorithms were designed and implemented^9,25,58^ to reach O(N) scaling of the time for Kohn–Sham matrix formation and evaluation of nuclear gradients. The resulting code can treat molecular and periodic systems of any dimension on an equal footing. riper includes also a new, low-memory modification of RI in combination with CFMM and a preconditioned conjugate gradient solver.^88^ Compared with the standard RI implementation, it allows for up to 15-fold reduction of the memory requirements at a cost of only a small increase in computation time. This has enabled DFT calculations for molecular systems with thousands of atoms on a single central processing unit (CPU) workstation.^88^

### G. Relativity and heavy elements

For heavy elements, relativistic effects are important for quantitatively and often even qualitatively correct results.^89^ These can be incorporated into the non-relativistic machinery by effective core potentials (ECPs) or relativistic all-electron approaches including arbitrary-order Douglas–Kroll–Hess (DKH) theory, Barysz–Sadlej–Snijders (BSS) Hamiltonian, and exact two-component (X2C) theory as these approaches only affect the one-electron part.^90^ A (modified) scaled nuclear spin–orbit (SNSO) approximation^91,92^ was implemented to account for the spin–orbit effects on the two-electron integrals.^93^ The all-electron approaches are available with a finite nucleus model based on a Gaussian charge distribution^94^ for the scalar^93^ and the vector potential.^95^ This accounts for the finite charge distribution of heavy elements and leads to a faster convergence of the energy with respect to the basis set limit.^96^ The X2C Hamiltonian should be used in all-electron calculations both from a conceptional point of view and for accuracy as low-order DKH can yield large errors, and the sequential decoupling becomes demanding for large molecules.^97^

The majority of today’s relativistic calculations still use ECPs, which approximate the core electrons and relativistic effects by a pseudopotential. Thus, the additional effort to include special relativity is reduced to simple one-electron integrals, which are available at essentially no extra cost. However, ECPs cannot be used for the excitation of core-electrons or properties that are driven by the density in the core region such as NMR shifts and coupling constants. Moreover, relativistic ECPs are typically only available for heavier elements than krypton.^98^ Hence, all-electron approaches are necessary to treat all elements and electrons on an equal footing. Relativistic all-electron approaches are computationally more demanding than ECPs due to the block-diagonalization of the Dirac Hamilton matrix and the comparably large basis sets. This is even more pronounced since the basis sets are employed in an uncontracted fashion during the decoupling as the contraction coefficients of the large and small component differ significantly. The contraction is then performed after the decoupling. Hence, local decoupling approaches were additionally implemented in TURBOMOLE to enable routine calculations of large molecules.^90,93,95^ As a result, the two-electron part becomes more time consuming than the relativistic one-electron terms—just as in the non-relativistic approach.

Relativistic effects are partitioned into scalar and spin–orbit contributions. Scalar-relativistic approaches can be readily introduced into the existing infrastructure of the one-component (1c) ansatz, whereas the latter break spin-symmetry and thus necessitate a generalized HF or (non-collinear) KS ansatz based on a two-component (2c) formalism. Moreover, spin–orbit (SO) coupling leads to imaginary operators in addition to the real scalar-relativistic ones. Thus, complex algebra and generalized solvers for response theory are needed. Additionally, error-consistent integration grids for the XC terms are available as the default grids were usually designed for light elements.^99^ Two-component calculations are available for energies,^29,100–103^ gradients,^93,102^ energy decomposition analysis (EDA), excited-state related properties such as ultraviolet–visible (UV/vis) spectra,^104,105^ *GW* methods,^106,107^ the Bethe–Salpeter equation (BSE),^33,108^ RPA,^109,110^ CC2,^111,112^ or symmetry-adapted perturbation theory (SAPT).^113^ The relativistic code exploits the same features as the non-relativistic machinery.

### H. Basis sets

From TURBOMOLE’s beginnings, code development was accompanied by basis set development. An overview with typical applications for the different basis set families developed with TURBOMOLE is given in Table I. The TURBOMOLE or Karlsruhe basis set family currently consists of four types. The now outdated “def” basis sets^20,114–116^ served as a very reasonable starting point for the development of a second generation of basis sets, the so-called “def2” bases.^117^ These def2 bases were designed to yield similar errors for ground-state properties all across the *s*, *p*, and *d* elements of the periodic table and later^118^ extended to *f* elements. Relativistic two-component treatments necessitate tailored basis sets. Starting from the def2 system, bases were optimized for the use together with one- and two-component Dirac–Hartree–Fock (DHF) effective core potentials for Rb–Rn, “dhf,”^119^ and later also for relativistic all-electron theories such as X2C.^120^ The interested reader is referred to the supplementary material.

**TABLE I.** Overview of the Karlsruhe basis set families. The accuracy is stated by the mean absolute errors in the atomization energy (per atom) in kJ/mol at the DFT level for the test set described in Ref. 117. The standard deviation is listed in parenthesis. Three types of auxiliary basis sets are considered: RI-*J* and RI-*K* at the DFT/HF level and auxiliary bases for post-HF/post-KS methods (see the supplementary material). Extensions for polarization effects (P), spin–orbit coupling (-2c) polarizabilities (D), and NMR shifts (-s) are described in the supplementary material.

	Accuracy						
Family	SV(P)	TZVP	QZVP	Key features and intended use	Elements	Auxiliary basis sets	Extensions
def	7.7 (13.4)	10.9 (10.7)	…	Obsolete	H–Rn (no 4*f*)	RI-*J*, RI-*K*	P
						Post-HF/post-KS	
def2	11.8 (9.7)	3.8 (2.6)	1.0 (1.0)	General one-component calculations	H–Rn	RI-*J*, RI-*K*	P, D
				Electric properties, dispersion/noncovalent		Post-HF/post-KS	
				Application to post-HF and post-KS			
dhf	11.0 (9.5)	2.9 (1.9)	0.4 (0.4)	Similar to def2 but consistent DHF ECPs	H–Rn (no 4*f*)	RI-*J*, RI-*K*	P, -2c
				Correction of small deficits of def2			
				Two-component for heavier elements			
x2c	6.2 (4.7)	1.9 (1.3)	…	Relativistic all-electron, finite nucleus model	H–Rn	RI-*J*	P, -2c, -s
				Core-region accessible, two-component for all			

### I. Parallelization

TURBOMOLE supports various parallel implementations designed for different needs. An overview of the available implementations is given in Table II. For calculations on a single node with possibly dozens of CPU cores, a shared-memory parallelization (SMP) using the OpenMP^121^ model has been implemented for almost all modules.^22,26,39,40,42^ The somewhat older Fork-SMP^41^ is still available as a fallback and not used by default. The parallel versions cover one- and two-electron integral routines (evaluation and contraction) and the XC part (grid generation and numerical integration) as well as linear algebra operations (solver for eigenvalue problems, Cholesky decomposition, matrix inversion, and sparse and dense basis transformations).

**TABLE II.** Available parallelizations for various modules. Fork-SMP^41^ and the OpenMP version^22,26,39,40,42^ are restricted to calculations on a single node, whereas MPI^35–38,122–124^ and OpenMP/MPI hybrid^125^ implementations allow for the use of multiple nodes. A detailed description of the modules is given in TURBOMOLE’s manual.^43^

Module	Fork-SMP	OpenMP	MPI	OpenMP/MPI
dscf	✓	✓	✓	✓
grad	✓	✓	✓	✓
aoforce	✓	✓	✓	✓
ridft	✓	✓	✓	X
rdgrad	✓	✓	✓	X
escf	✓	✓	✓	✓
egrad	✓	✓	X	X
mpgrad	✓	X	✓	X
mpshift	X	✓	X	X
ricc2	X	✓	✓	✓
pnoccsd	X	✓	✓	✓
ccsdf12	X	✓	X	X
rirpa	X	✓	X	X
riper	X	✓	X	X

In most HF and DFT modules, OpenMP uses atomic updates to sum up the contribution to the target matrices such that they do not have to be replicated for each thread and the required memory does not increase much with the number of threads used. This is especially important for the calculation of ground-state vibrational frequencies and excited-state gradients, where a large number of response equations solved simultaneously produces a high memory demand. The reduction clauses are only used to accumulate scalars or small vectors such as the nuclear geometry gradient. Parallelization of the integral calculation is done by distributing batches of integrals to individual workers (threads), while in the numerical quadrature (XC), batches of about 100 grid points are used to distribute the work.

If one node is not powerful enough, one can use a distributed memory parallelization based on the message passing interface (MPI)^126^ library. The preferred mode of operation is then a OpenMP/MPI hybrid scheme^125^ in which one MPI process is run on each node using all of the CPU cores available there via OpenMP. This mode is most memory-economic because the MPI parallelization often involves replicated data in each of the MPI processes. An equal distribution of work load among the MPI processes is a critical issue in distributed memory parallelization. For post-Hartree–Fock methods, the work associated with individual tasks can be faithfully estimated beforehand, and this allows for a static distribution of the load.^38^ For Hartree–Fock and especially for the numerical quadrature tasks performed in the DFT modules, a dynamic load balancing scheme has been chosen^125^ in which a lightweight server task distributes all the work load to the compute tasks upon their request. The somewhat older MPI-based parallelization of the ridft module and rdgrad module^122,123^ was reworked recently and exploits distributed shared memory, dynamic load balancing without the need for server tasks, thread-based parallel linear algebra, and non-overlapping shared memory access.^124^

## III. SELECT EXAMPLES OF TURBOMOLE’S FUNCTIONALITY

### A. Thermochemistry

For accurate calculations of electronic contributions to reaction energies, intermolecular interactions, and spin splittings, explicitly correlated coupled-cluster methods offer an excellent cost to performance ratio due to enhanced basis set convergence. The ccsdf12 program offers all state-of-the-art explicitly correlated CCSD variants, including CCSD(F12), CCSD(F12^*^), ${\text{CCSD}}_{\left({\text{F}12}^{*}\right)}$, CCSD[F12], CCSD-F12b, CCSD-F12a, ${CCSD\left(2\right)}_{\bar{F12}}$, and the Brueckner coupled-cluster method ${\text{BCCD}}_{\left({\text{F}12}^{*}\right)}$. Each can be combined with the conventional triples correction (T) or the scaled triples correction (T^*^). CCSD(F12^*^), which in the literature is sometimes also denoted as CCSD-F12c, is usually the method of choice since it outperforms the other low cost CCSD-F12 variants in terms of accuracy.^85,127,128^

**FIG. 1.**
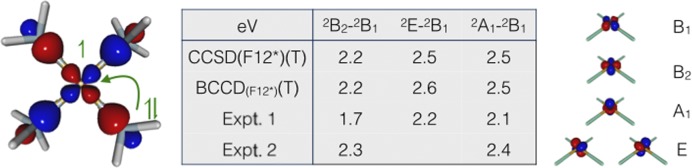
Ligand to metal charge transfer in Cu(NH_3_)_2+_^4^ and 3*d*–3*d* transition energies in eV.^129^ Reprinted (adapted) with permission from Giner *et al.*, J. Chem. Theory. Comput. **14**, 6240–6252 (2018). Copyright 2018 American Chemical Society.

Figure 1 reports the lowest three electronic transitions of the Cu(NH_3_)^2+^_4_ complex in the gas phase taken from Ref. 129 computed using the CCSD(F12^*^)(T) and ${\text{BCCD}}_{\left({\text{F}12}^{*}\right)}$(T) methods and compared to values from two sets of polarized single-crystal electronic spectra: Expt. 1 (Ref. 130) and Expt. 2 (Ref. 131). The Brueckner coupled-cluster is important for transition metal complexes, where the HF orbitals relax substantially upon correlation, and has recently been implemented in the ccsdf12 program for open and closed-shell systems.^132^ The four electronic states ^2^B_2_, ^2^B_1_, ^2^A_1_, and ^2^E differ in the vacant orbital of the 3*d*^9^ configuration at the copper center and are ground states in their respective *D*_2*d*_ point group irreducible representation. Although only *D*_2*h*_ subgroups can be exploited in CCSD/BCCD calculations and no symmetry is yet available in F12 calculations, HF references of arbitrary symmetry can be used by converting orbitals between symmetries using the define module. The ECP, DKH, BSS, and X2C one-component relativistic treatments are all available in combination with F12 calculations. In this example, the aug-cc-pwCVTZ-DK basis sets^133–136^ are used with the X2C relativistic Hamiltonian^90^ and all but the 1*s*2*s*2*p* orbitals on copper are correlated. The relative stabilities of the electronic states are governed by ligand field splitting and differing levels of stabilization from ligand to metal charge transfer. Both sets of coupled-cluster calculations agree with the transitions observed for the crystal structure in experiment 2 and confirm the hypothesis that the crystal structure measured in experiment 1 does not correspond to a square-planar Cu–N_4_ motif.

For single-reference systems, CCSD(F12^*^)(T) calculations with a triple-*ζ* basis typically provide reaction energies accurate to 1 kcal/mol,^128^ structures accurate to 1 pm,^137^ and vibrational frequencies accurate to 5 cm^−1^.^127^ For higher accuracy, CCSD(F12^*^) energies computed using the ccsdf12 program with quadruple-*ζ* or higher basis sets can be combined with higher-order correlation treatments from other programs.^138^ Friedrich and co-workers used CCSD(F12^*^)(T) in combination with an incremental scheme^139,140^ to calculate accurate reaction and interaction energies for large molecules and established accurate benchmark data for reaction energies. Their incremental scheme is not part of the TURBOMOLE software suite, but TURBOMOLE offers low scaling explicitly correlated CCSD methods based on the pair natural orbital approach.^141^

**FIG. 2.**
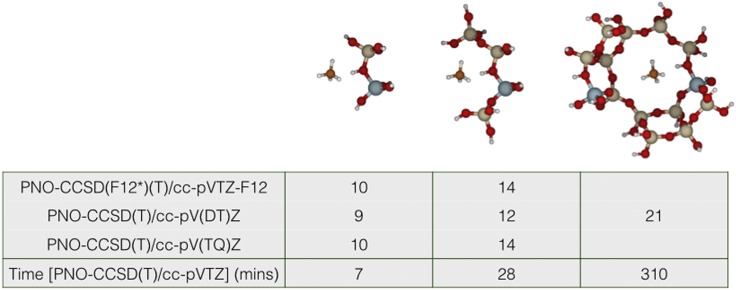
Interaction energies of methane with molecular models of H-chabazite in kJ/mol.

The pnoccsd program provides an O(N) implementation of PNO-CCSD(T) for ground-state energies of open and closed-shell molecules [$O\left(N^{3}\right)$ for PNO-CCSD(F12^*^)(T) at the time of writing this article], which exploits the sparsity in the wavefunction parameters and Hamiltonian matrix elements resulting from the rapid decay of correlation between increasingly distant electrons in insulators. Accurate interaction energies for sizable systems can be computed in a few hours. Figure 2 reports the interaction energy of methane with three successively larger cluster models of the zeolite H-chabazite (Al_1_Si_1_O_7_H_7_, Al_1_Si_3_O_13_H_13_, and Al_2_Si_12_O_38_H_22_ taken from Ref. 142) computed using the pnoccsd program. Energies are listed for PNO-CCSD(F12^*^)(T) using the cc-pVTZ-F12 basis^143^ and for PNO-CCSD(T) using extrapolated counterpoise corrected energies to approach the basis set limit with cc-pV*X*Z (*X* = D, T, Q) basis sets^133,144^ and Helgaker’s two-point extrapolation.^145^ The PNO threshold was 10^−7^ in all calculations, and the PNO truncation errors are sub-kJ/mol in the interaction energy. The largest calculation with 79 atoms and 2010 basis functions took only 5 h to run on a 40 core Intel Xeon Gold 6138 CPU @ 2.00 GHz machine.

### B. Noncovalent interactions of large molecules

**FIG. 3.**
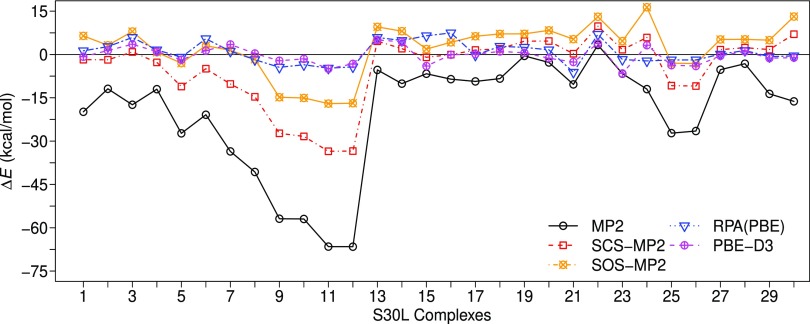
S30L interaction energy errors (Δ*E*) for MP2 variants, RPA(PBE), and dispersion corrected PBE-D3. Reference binding values are based on DLPNO-CCSD(T).^146^ A positive error corresponds to underbinding. Reprinted with permission from Nguyen *et al.*, J. Chem. Theory Comput. **16**, 2258–2273 (2020). Copyright 2020 American Chemical Society.

**FIG. 4.**
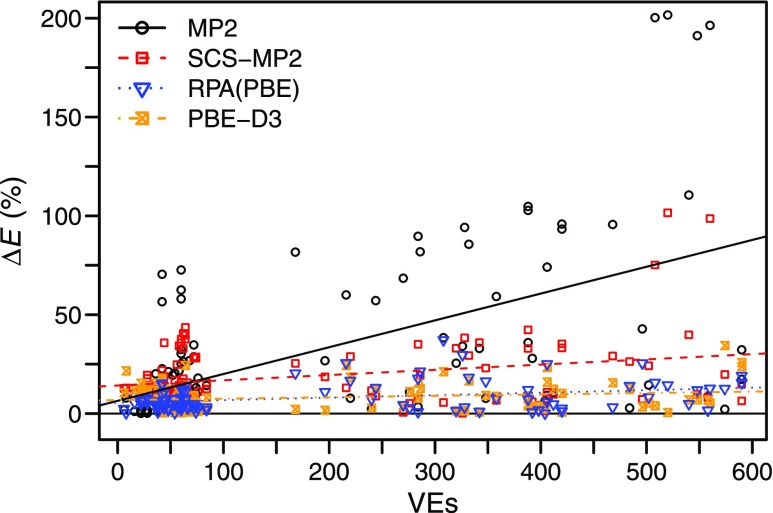
Relative errors (Δ*E*) of MP2 variants, RPA with a PBE^154^ Kohn–Sham reference [RPA(PBE)], and PBE with D3 dispersion correction (PBE-D3)^152^ for interaction energies in the S66,^155,156^ L7,^157^ and S30L^158^ benchmarks vs number of valence electrons (VEs). Reprinted with permission from Nguyen *et al.*, J. Chem. Theory Comput. **16**, 2258–2273 (2020). Copyright 2020 American Chemical Society.

Noncovalent interactions (NIs) play essential roles in molecular biology and supramolecular chemistry.^147^ Until recently, many-body perturbation theory (MBPT) has been the method of choice to predict NIs of molecules as they have been considered as “weak interactions.” However, an increasing number of examples report substantial overestimation of NI energies for MP2.^148,149^ This is also evident from the computed MP2 binding energies for the S30L complexes in Fig. 3. Other variants of MP2 such as the spin-component-scaled MP2 (SCS-MP2)^150^ and scaled opposite-spin MP2 (SOS-MP2)^151^ performed poorly as well. On the other hand, RPA maintains high accuracy for NIs of large molecules on par with the dispersion corrected DFT methods. This result is remarkable since RPA is free of empirical adjustments as opposed to the dispersion-corrected DFT^152,153^ and empirically scaled MP2 methods. Relative errors in NIs of MP2 and SCS-MP2 grow linearly with the system size, whereas those of RPA and dispersion-corrected DFT stay virtually constant, as shown in Fig. 4 and Table III.

**TABLE III.** Parameters of the linear regression fits displayed in Fig. 4. The slope corresponds to the average relative interaction energy error per valence electron (VE), and the *y*-intercept corresponds to the average relative interaction energy error in the limit of zero VEs. Reprinted with permission from Nguyen *et al.*, J. Chem. Theory Comput. **16**, 2258–2273 (2020). Copyright 2020 American Chemical Society.

Method	Slope (%/VE)	*y*-intercept (%)
MP2	0.1219	7.30
SCS-MP2	0.0251	14.10
RPA(PBE)	0.0030	6.24
PBE-D3	0.0083	6.79

An asymptotic adiabatic-connection symmetry-adapted perturbation theory (AC-SAPT) has been developed to examine NIs between molecules at full coupling under a density constraint.^159^ The AC-SAPT expansion for finite-order MBPT may diverge especially for large or polarizable molecules, whereas the AC-SAPT expansion for RPA is always convergent.^159^ Consequently, the assumption that NIs are weak compared with covalent interactions is incorrect, and finite-order MBPT is generally inaccurate for NIs, except for small systems with a low polarizability. RPA, on the contrary, is accurate for a wide range of applications independent of system size, gap size, or empirical training sets.^159^ RPA may safely replace MP2 for calculations of NIs in most systems of chemical interest, provided proper auxiliary basis sets are used for the RI approximation in RPA.^160^ The RPA implementation in TURBOMOLE can readily compute the NI energy between pentakis(1,4-benzodithiino)corannulene and C_60_ in a 1:1 complex with 140 atoms within a day, as shown in Table IV.

**TABLE IV.** Total computational wall time (min) and energy consumption (kWh) for the RPA binding energy using a PBE Kohn–Sham reference^154^ and cc-pVTZ basis sets^133,144^ for a 140-atom fullerene catcher complex with pentakis(1,4-benzodithiino)corannulene being the host and C_60_ being the guest.^158^ 100, 60, and 160 core orbitals were frozen for the RPA calculations on the host, the guest, and the complex, respectively. The calculations were performed on an Intel Xeon CPU E5-2680 v2 with 10 cores and 50 Gb RAM using the ridft and rirpa modules.

	DFT		RPA	
Molecule	*t* (min)	Energy (kWh)	*t* (min)	Energy (kWh)
Complex	54	1.04	807	15.47
Host	8	0.15	109	2.09
Guest	7	0.13	71	1.36

### C. Charge localization/delocalization in mixed-valence systems

Mixed-valence (MV) systems feature two or more electronically coupled redox centers with different (formal) oxidation states, which make them important models for understanding electron-transfer processes. Often, it has to be decided if spin and charge densities of a given MV system are partly localized to one center or fully delocalized over all of them. Among the many challenges for computations,^161^ finding a balance between avoiding delocalization errors and properly simulating left–right correlation in bonds is often decisive. A widespread computational protocol is based on global hybrid functionals with elevated exact-exchange (EXX) admixtures (∼35%–40%).^161,162^

While the first implementation of local hybrid functionals (local hybrids) in TURBOMOLE employing a seminumerical exchange approximation dates back to 2012,^101^ the SCF^163^ and ground-state gradient^164^ implementations of local hybrids^165^ have allowed the evaluation of this class of functionals for the structures and energetics of MV systems. The functional form of local hybrids is discussed in detail in a recent review,^165^ and we merely stress here that the position-dependent EXX admixture (via a local mixing function) offers additional flexibility for dealing with the balance between left–right correlation and delocalization errors in DFT, which is crucial for MV systems.

**FIG. 5.**
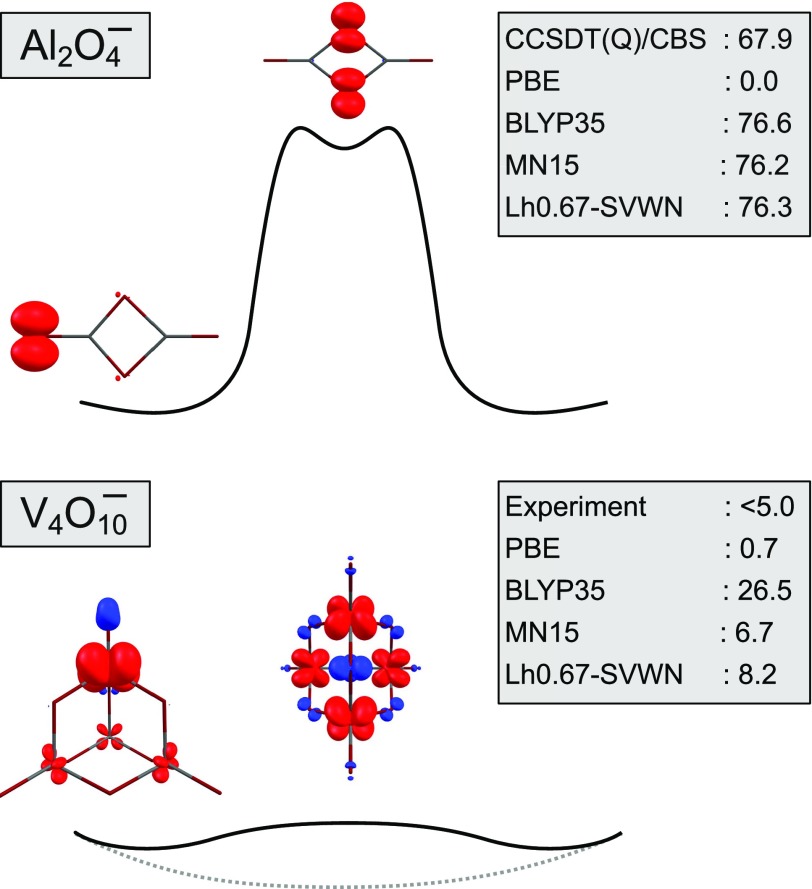
Spin-density distributions (±0.01 a.u. isosurfaces) of ${Al}_{2}O_{4}^{-}$ and $V_{4}O_{10}^{-}$ at minimum structures (left) and a transition state/high-lying minimum (right) calculated at the *ω*B97X-D^169^/def2-TZVP level. Relative energies at different levels of theory are given in kJ/mol. CBS denotes the complete basis set limit. The drawings give a qualitative impression of the potential energy curves (dashed line for a delocalized structure). The *D*_2*d*_ structure of the $V_{4}O_{10}^{-}$ transition state was deduced from the experimental spectra (see Ref. 166 for details). Reprinted (adapted) with permission from Klawohn *et al.*, J. Chem. Theory Comput. **14**, 3512–3523 (2018). Copyright 2018 American Chemical Society.

In the comparison of a wide range of XC functionals for a benchmark set of small gas-phase MV oxide systems (MVO-10),^166^ a local hybrid and a highly parameterized global hybrid (MN15^167^) exhibited the best balance for simultaneously describing the most localized and delocalized systems correctly. It is sufficient to focus here on the two most extreme cases, the oxyl-centered localized ${Al}_{2}O_{4}^{-}$ radical anion and the fully delocalized metal-centered $V_{4}O_{10}^{-}$. In Fig. 5, the spin-density plots of these systems are given for their minimum structures as well as for the respective transition state that connects two symmetry equivalent minima. We note that for ${Al}_{2}O_{4}^{-}$, the best calculations suggest the presence of a high-lying shallow minimum with bridge-localized spin density on top of the barrier, which is not reproduced by functionals with significant delocalization errors such as PBE (relative energy indicated as 0.0 kJ/mol in Fig. 5).^168^

Of those of the many functionals studied (four have been selected for Fig. 5) that reproduce the localized situation and the high CCSDT(Q) barrier for ${Al}_{2}O_{4}^{-}$, none obtain a properly delocalized *D*_2*d*_ structure for $V_{4}O_{10}^{-}$. However, the stabilization of a localized *C*_*s*_ structure for the latter system was found to be smallest with the highly parameterized MN15 global hybrid and with the simple one-parameter Lh0.67-SVWN local hybrid. These were the only functionals in that study that gave the correct localized structure for the aluminum radical anion and a barrier of less than 10 kJ/mol for the vanadium system, while other functionals that get the aluminum system right will stabilize a distorted vanadium anion more strongly.^166^ Meanwhile, improved local hybrids based on calibrated exchange-energy densities^170,171^ and with more sophisticated correlation functionals have been constructed, which will become available in the next release of TURBOMOLE (included in commit ID 33fd074d, tag V7-5-initial, January 22, 2020).

### D. Low-valent rare-earth and actinide coordination compounds with unconventional electronic structure

Rare-earth (Ln) and actinide (An) complexes are of scientific and industrial interest for their role in magnetism,^172–174^ small molecule activation,^175–177^ and nuclear fuel and waste processing.^178^ Computational studies of such complexes are crucial to their understanding due to the experimental challenges in their accessibility, handling, and characterization. However, the near degeneracy of the *f* and possibly *d* valence orbitals (*vide infra*) necessitates a balanced treatment of static and dynamic correlation, while the presence of bulky and oftentimes tailor-designed ligands requires methods with low computational cost. The situation is further complicated by solvation and relativistic effects. Although high-level wavefunction methods have been used to study small compounds or simplified model systems,^179–181^ DFT and post-KS methods remain the primary workhorse for routine computations of rare-earth and actinide complexes with large ligands.^182^

The state-of-the-art DFT and TDDFT implementations in TURBOMOLE have been shown to be well suited for predicting the electronic structure and properties of rare-earth and actinide complexes. For example, in 2009, calculations showed significant lanthanide 5*d* orbital occupations in a formal (N_2_)^3−^ complex of Dy.^175^ This observation inspired collaborative experimental and computational investigations to isolate reductive divalent lanthanide complexes with valence *d* populations, leading to the discovery of the first molecular complexes containing formal +2 oxidation states for the rare-earth metals Sc,^183^ Y,^184^ Ho,^185^ Er,^185^ Pr,^186^ Gd,^186^ Tb,^186^ and Lu^186^ and for the actinide metals Th,^187^ U,^188^ and Pu.^189^ The valence *d* orbitals in these compounds are stabilized by a trigonal field of substituted cyclopentadienyl ligands^190^ or amide ligands.^183,191^ The unique electronic structure of these complexes gives rise to unusual chemical reactivity, such as forming metal–metal bonds^192^ and reduction of CO, CO_2_, and N_2_.^183,193,194^ Prominent *d* occupations are also shown to be present in bis(cyclopentadienyl) Ln(II)^195^ and An(II)^196–198^ complexes, where the $d_{z^{2}}$ orbital participates in covalent *σ*-bonding, leading to linear coordination geometry. The lanthanocenes, in particular, afford high blocking temperature and slow magnetic relaxation while maintaining high magnetic anisotropy through the 4*f* electrons,^195^ demonstrating great potential as single-molecule magnets.

**FIG. 6.**
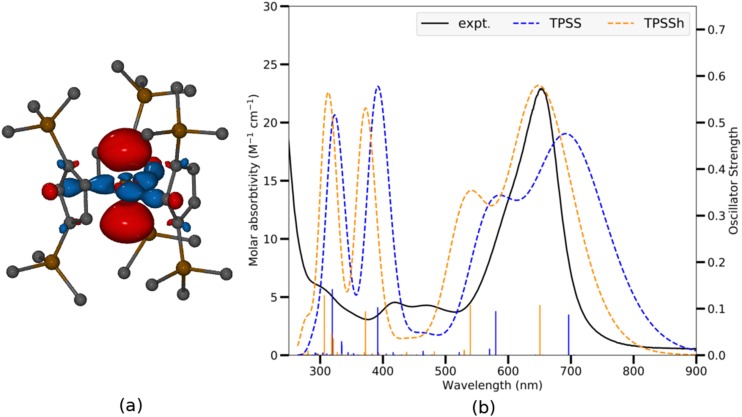
(a) Molecular orbital contour for $6d_{z^{2}}$ HOMO of {$Th{\left[C_{5}H_{3}{\left({SiMe}_{3}\right)}_{2}\right]}_{3}$}^−^ at 0.05 isovalue. Hydrogen atoms are omitted for clarity. (b) Experimental and TDDFT simulated UV/vis spectra of {$Th{\left[C_{5}H_{3}{\left({SiMe}_{3}\right)}_{2}\right]}_{3}$}^−^ using the TPSS and TPSSh functional. A universal blue shift of 0.25 eV was applied.

Table V showcases the speed and energy consumption of DFT and post-KS calculations for {Th(II)${\left[C_{5}H_{3}{\left({SiMe}_{3}\right)}_{2}\right]}_{3}$}^−^ (Fig. 6), an *f*-block-element compound with transition-metal-like 6*d*^2^ ground state.^187^ Particularly for the Th(II) complex, the qualitatively correct TDDFT results compared with the solution-phase UV/vis spectrum played an important role for the characterization of the Th 6*d*^2^ configuration, where the strong absorption in the visible region was assigned to excitations from the valence $d_{z^{2}}$ orbital, as was also shown for its Ln(II) and U(II) analogs.^188,199^ To correct for intrinsic problems of semilocal DFT such as self-interaction error and the lack of noncovalent interactions, RPA and beyond-RPA methods within the rirpa module provide a good balance between computational cost and accuracy.^13,200,201^ The hierarchy of semilocal density functional approximations, RPA, and the beyond-RPA approximate exchange kernel (AXK) method^13,202^ provide a systematic way of computing and validating the DFT ground-state energy for systems with weak to moderately strong correlation.The suitability of RPA and AXK for a particular system is measured by an effective coupling strength measure $\bar{\alpha }$ as defined in Ref. 13, where a smaller $\bar{\alpha }$ value corresponds to weaker correlation and better accuracy of RPA and AXK. For {$Th{\left[C_{5}H_{3}{\left({SiMe}_{3}\right)}_{2}\right]}_{3}$}^−^, the energy difference between the triplet 5*f*^1^6*d*^1^ state and the singlet 6*d*^2^ state is computed to be 10 kcal/mol, 19 kcal/mol, and 22 kcal/mol using the semilocal TPSS functional,^203^ RPA, and AXK, respectively. The effective coupling strength $\bar{\alpha }$ values are ∼0.43 for both the 5*f*^1^6*d*^1^ and the 6*d*^2^ states, indicating that the error of the AXK energy difference is ≲ 10 kcal/mol^13^ and confirming the 6*d*^2^ ground state.

**TABLE V.** Total computational time (min) and energy consumption (kWh) for DFT single point, analytic Hessian, TDDFT, and AXK calculations on {$Th{\left[C_{5}H_{3}{\left({SiMe}_{3}\right)}_{2}\right]}_{3}$}^−^ using TPSS and TPSSh functionals. 72 core orbitals were frozen for the AXK calculation. The calculations were performed on an Intel Xeon Gold 6148 @ 2.40 GHz CPU with 16 cores and on an AMD Ryzen 9 3900X @ 3.8 GHz CPU with 12 cores.

		Intel		AMD	
			Energy		Energy
Calc.	Functional	*t* (min)	(kWh)	*t* (min)	(kWh)
DFT^a^	TPSS	3	0.01	2	0.01
	TPSSh	8	0.02	7	0.01
Hessian^b^	TPSS	270	0.68	197	0.34
	TPSSh	640	1.60	502	0.88
TDDFT^c^	TPSS	215	0.54	181	0.32
	TPSSh	1823	4.56	1880	3.29
AXK^d^	TPSS	166	0.42	307	0.54

^a^ Single-point DFT calculation using the ridft module with the def-TZVP basis set on the Th atom and the def2-SV(P) basis set on the C, H, ans Si atoms, totaling 795 basis functions. The Stuttgart/Cologne ECP was used for Th.^204^

^b^ Force constant calculation for vibrational normal modes using the aoforce module with the same basis as the ridft calculations.

^c^ TDDFT calculation for the lowest 60 excitations using the escf module with the def-TZVP basis set on the Th atom and the def2-SVPD basis set on the C, H, and Si atoms.

^d^ RI-AXK calculation using the rirpa module with the def-TZVP basis set on the Th atom, the def2-TZVP basis set on the ring C atoms, and the def2-SV(P) basis set on the remaining C, H, and Si atoms.

### E. Accurate reaction barriers

**FIG. 7.**
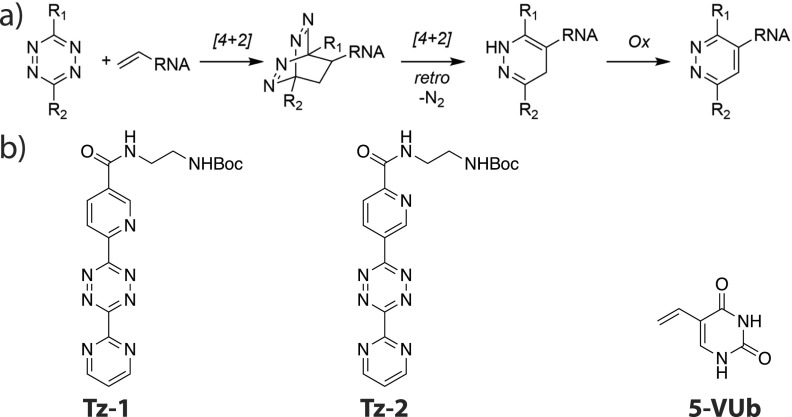
(a) Schematic of the inverse electron-demand Diels–Alder reaction with vinyl-modified RNA. (b) Tetrazines (**Tz-1** and **Tz-2**) and vinyl-nucleoside (**5-VUb**) discussed in Sec. III E. Reprinted with permission from Kubota *et al.*, ACS Chem. Biol. **14**, 1698–1707 (2019). Copyright 2019 American Chemical Society.

The broad range of available functionalities allows for the comprehensive exploration of reaction profiles for reactions with biological and industrial relevance. Kubota *et al.*^205^ recently designed a new chemical method to tag nascent RNA through application of inverse electron-demand Diels–Alder (IEDDA) chemistry and demonstrated its ability to tag and image RNA in cells. Vinyl-modified RNA was tagged with a tetrazine derivative, as shown in Fig. 7(a). Optimal tetrazine–vinyl-nucleoside pairs—with fast reactivity and low toxicity—were determined with the help of reaction profiles computed using the TPSS density functional. Because the tetrazines studied are flexible, equilibrium geometries and transition states are partially stabilized by long-range interactions, and the reactivity is highly sensitive to the solvent, the computational model needed to carefully balance competing effects of dispersion, solvation, and enthalpic and entropic vibrational contributions.^205^ Furthermore, due to the partial symmetry of the tetrazines and vinyl-nucleosides, many reaction profiles with similar free energies had to be considered, meaning computational efficiency was paramount. The reaction rates estimated with DFT consistently reproduced the experimental observations even for challenging cases that are poorly predicted by simple models based on driving forces computed from frontier orbital energies. For example, when reacting with vinyl nucleoside **5-VUb** (see Fig. 7(b), the tetrazine **Tz-1** reacts faster than **Tz-2** by a factor of 10; DFT predicts a ratio of 10.1:1, whereas the frontier orbital driving force predicts no difference in kinetics.^205^

**FIG. 8.**
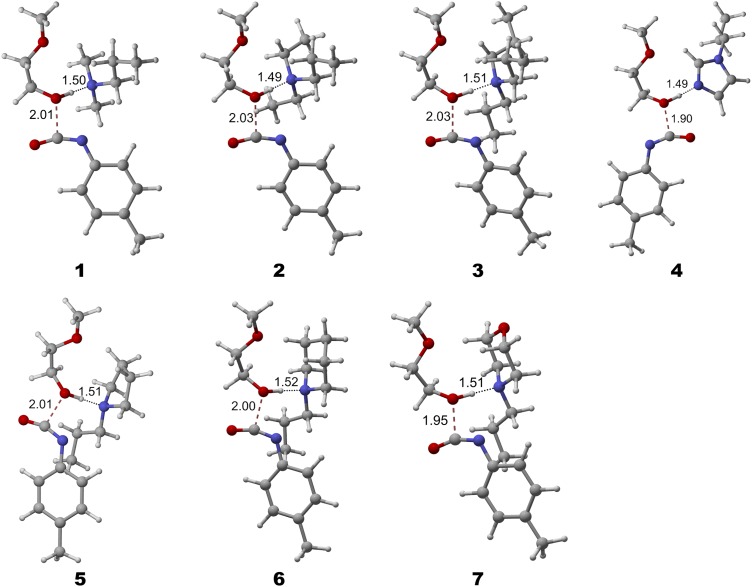
Transition states of tertiary amide catalyzed urethane reactions between an aromatic isocyanate and a model polyol. Reprinted with permission from Muuronen *et al.*, J. Org. Chem. **84**, 8202–8209 (2019). Copyright 2019 American Chemical Society.

Muuronen *et al.*^206^ used TURBOMOLE to predict the catalytic activity of tertiary amides for synthesizing polyurethanes and confirmed their prediction experimentally. Polyurethanes are commonly used in the manufacture of everyday products such as furniture and shoe soles. Traditional catalysts with *N*,*N*-dimethyl groups may be oxidized when exposed to air and emit formaldehyde, an indoor air pollutant. Similar to the IEDDA example, the computational design of new polyurethane catalysts without formaldehyde emission required a balanced treatment of electron correlation, dispersion, solvation, and thermal effects. In particular, the effect of conformational entropy^207^ turned out to be important to rationalize the trend of catalyst activities of amides with various alkyl chain lengths and ring sizes. The relative activities of the candidate catalysts (Fig. 8) from experimental kinetic measurements were in accordance with the computed activation free energies at the RPA level of theory. The RPA calculations were also corroborated by the beyond-RPA perturbation correction using the AXK method.^13,202^

### F. Periodic systems and materials

One of the newer features of the TURBOMOLE program is the module riper, which allows users to perform DFT calculations applying periodic boundary conditions. Its key component is a combination of the RI approximation and CFMM used for rapid evaluation of the electronic Coulomb term.^9,25^ The calculation of the exchange–correlation contribution to energy and nuclear gradients employs a hierarchical numerical integration scheme^58,208^ and a robust periodic Fock exchange implementation.^209^ Recently, the calculation of stress tensor and energy first derivatives with respect to lattice vectors has also been implemented.^210^

**FIG. 9.**
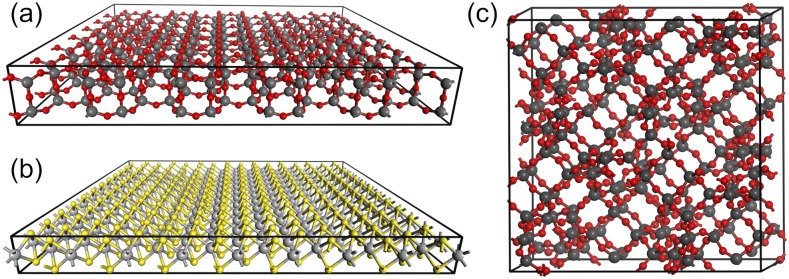
Benchmark systems with 2D and 3D periodicity: (a) 5 × 7 unit cell of 2D SiO_2_,^213^ (b) 8 × 12 unit cell of 2D MoS_2_, and (c) unit cell of 3D faujasite zeolite (FAU) (O, red; S, yellow; Si, dark gray; and Mo, light gray).

Thanks to its flexible implementation, riper can treat molecular and periodic systems of any dimensionality on an equal footing. In contrast to the commonly used plane wave basis sets, the use of Gaussian basis functions allows treating one- (1D) and two-dimensional (2D) systems without the need for constructingartificial models with three-dimensional (3D) periodicity. As an example, riper was used to determine the complex mechanism of a Stone–Wales type defect formation in two-dimensional SiO_2_.^213^ The module is also particularly well suited for DFT calculations on sparsely packed systems, such as zeolites and metal–organic and covalent–organic frameworks. For example, it was recently used to elucidate topochemical conversion of an imine-linked into a thiazole-linked covalent organic framework.^214^ Computational efficiency and favorable scaling behavior of the implementation approaching O(N) for the calculation of energy and nuclear gradients have been demonstrated for various molecular and periodic systems, with the largest one containing several thousands of atoms.^9,88,208,210^ Table VI shows examples of wall times for DFT calculations with different exchange–correlation functionals^154,215–218^ of various two- and three-dimensional systems shown in Fig. 9.

**TABLE VI.** Wall times (s) for the selected 2D and 3D systems. Number of atoms (*N*_at_), number of basis functions (*N*_bf_), time per SCF iteration (*t*_SCF_) for the Coulomb (*t*_J_) and XC (*t*_xc_) parts as well as the total time for calculation of nuclear gradient (*t*_grad_). The calculations used 36 cores of two Xeon Gold 6140 CPUs and 192 Gb RAM.

System^a^	Functional	*N*_at_	*N*_bf_	*t*_SCF_	*t*_J_	*t*_XC_	*t*_grad_
SiO_2_ (2D)	PBE	840	17 080	399	180	17	173
SiO_2_ (2D)	B3LYP	840	17 080	1609	185	1215	…^b^
MoS_2_ (2D)	PBE	576	15 936	323	144	13	151
MoS_2_ (2D)	M06-L	576	15 936	352	146	35	163
FAU (3D)	PBE	576	11 712	271	187	11	166
FAU (3D)	CAM-B3LYP	576	11 712	1120	174	880	…^b^

^a^ All calculations used pob-TZVP basis sets from Refs. 211 and 212.

^b^ Nuclear gradient not yet implemented.

### G. NMR and EPR spectra: Large molecules, heavy elements

Chemical shielding tensors at the Hartree–Fock, DFT, and MP2 level have been available in TURBOMOLE for a long time,^4,17,219–221^ including the exploitation of Abelian and non-Abelian point group symmetry.^4,17^ Recent additions^26,222^ enhance the functionality to the (gauge-origin invariant) use of ECPs based on Ref. 223, *meta*-GGA functionals, range-separated hybrid functionals, and COSMO to account for solvent molecules or counterions. Shorter computation times are achieved by more efficient integral screening for the exchange integrals and the possibility of nuclei selection, as well as the employment of the RI-*J* approximation and its multipole-accelerated variant,^27^ MARI-*J*, for HF and DFT.^26^

**FIG. 10.**
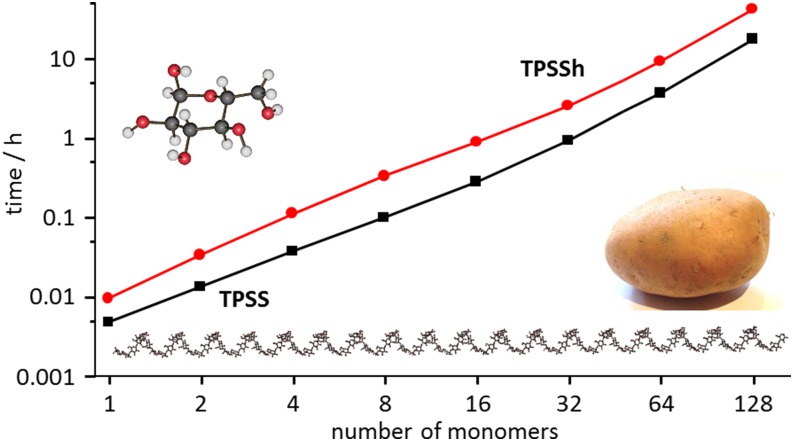
Computation times for the calculation of chemical shifts on chains with different numbers of glucose units, as reported in Ref. 26. Reprinted with permission from Reiter *et al.*, J. Chem. Theory Comput. **14**, 191–197 (2018). Copyright 2018 American Chemical Society.

The efficiency has been demonstrated for chains of *α*-D-glucose units in Ref. 26; some of the computation times are visualized in Fig. 10. The largest molecule therein, the 128-membered glucose chain, was recalculated using the release version 7.4 of TURBOMOLE on an Intel Xeon Processor E5-2687W v4 @ 3.0 GHz. The chemical shift calculations with a 6-31G^*^ basis set^224^ take 13 h and 33 h for TPSS^203^ and TPSSh,^203,225^ respectively, consuming 0.3 kWh and 0.7 kWh. In both cases, this is less time than for the calculation of the wavefunction (37 h consuming 0.7 kWh and 43 h consuming 0.9 kWh). The parallel (OpenMP) speed-up in the chemical shift calculations on four threads is 3.3 for both functionals. Further improvement is difficult to achieve because disk operations take a significant amount of time for such large systems.

**FIG. 11.**
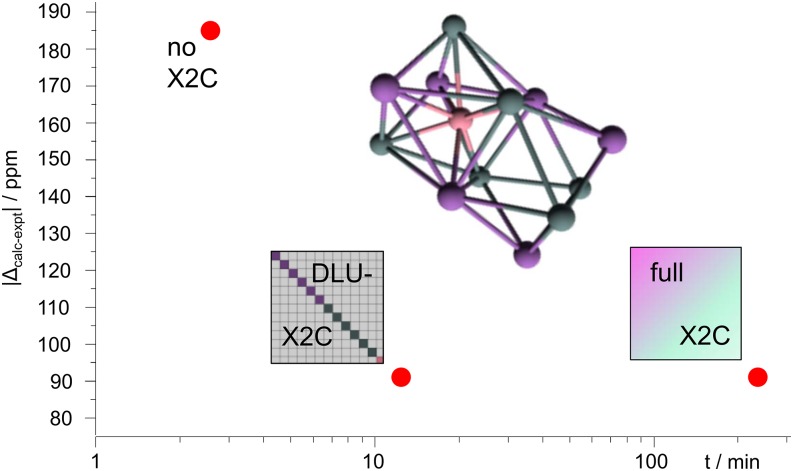
NMR shifts of endohedral 12-vertex clusters of Ref. 229. Comparison of deviation from experimental results and computation time of one-component all-electron approaches. The weighted average was calculated according to the Boltzmann distribution. The standard deviation is about 70 (NR) to ∼100 ppm (X2C). Reprinted (modified) with permission from Franzke and Weigend, J. Chem. Theory Comput. **15**, 1028–1043 (2019). Copyright 2019 American Chemical Society.

Furthermore, for calculations of NMR shifts of heavy elements, which involve a gauge-origin invariant scalar-relativistic exact two-component approach including a finite nucleus model for the scalar and the vector potential, the response of the relativistic decoupling matrix and the proper balance condition are implemented.^90,93,95,99^ Here, the scalar-relativistic one-electron Dirac-Hamiltonian in a basis set expansion is block-diagonalized based on a unitary transformation. To facilitate the calculation, the so-called diagonal local approximation to the unitary decoupling transformation (DLU),^226^ is available. There, the Dirac matrix is partitioned into atomic blocks, and thus, the dimension is reduced. This allows for routine calculations of molecular properties on workstation computers.^227^ The results and timings for the non-relativistic (NR), X2C, and DLU-X2C Hamiltonian are compared in Fig. 11. The error introduced by the DLU scheme is negligible but increases with diffuse functions. Its application results in a significant speed-up and low memory requirements, as only the small atomic blocks are considered.^95^ Hence, large heavy-element clusters with more than 6000 primitive basis functions can be treated with a total computation time of 8 h on an Intel Xeon Processor E5-2687W v2 @ 3.4 GHz using a single core (0.1 kWh). The DLU-X2C Hamiltonian and recently developed NMR-tailored basis sets^99^ were employed to analyze the NMR and UV/vis spectra of low-valent group 14 phosphinidenide complexes (with more than 100 atoms) and to rationalize the first *pπ*–*pπ* bond between phosphorus and lead.^228^

Developments for the next release version of TURBOMOLE (included in commit ID 33fd074d, tag V7-5-initial, January 22, 2020) include a new solver for linear response equations in the spirit of Refs. 10 and 65 and a simplified input for nuclear-independent chemical shifts (NICS).^230^ Furthermore, the (non-relativistic) calculation of nuclear spin–spin coupling constants will be extended to all terms. Using the MARI-*J* approximation for the Coulomb term together with semi-numerical integration techniques for HF-exchange integrals, the nuclear-shielding implementation has been extended to local hybrid functionals.^231^

Electron paramagnetic resonance (EPR) parameters for open-shell systems are influenced crucially by spin–orbit effects and described by a two-component formalism. The approach is presently available only in a local version of TURBOMOLE^232,233^ and presented in the supplementary material.

### H. Vibrational circular dichroism spectra

Recently, the program suite was extended to calculate vibrational circular dichroism (VCD) spectra at the Hartree–Fock and (hybrid) DFT level,^222^ which allows the identification of the absolute configuration of chiral molecules. This implementation mainly follows Cheeseman *et al.*^234^ and allows for the usage of effective core potentials to describe scalar-relativistic effects introduced by heavy elements. Both the vibrational frequencies and the magnetic response are needed for the calculation of VCD spectra, which are provided by the module for the calculation of vibrational frequencies (aoforce) and the NMR module (mpshift).

For VCD spectra, the same requirements concerning basis sets and functionals must be fulfilled as for the calculation of infrared (IR) spectra, since VCD and IR spectra only differ in their intensities, whereas the frequencies are the same. Most of the computation time is needed for the calculation of the frequencies; the additional effort for the calculation of the magnetic response is negligible and only amounts to 2%–5% of the total CPU time. Symmetry can be exploited^15,17^ to accelerate the calculations for molecules that belong to the chiral point groups *C*_*n*_, *D*_*n*_, *T*, *O*, or *I*.

**FIG. 12.**
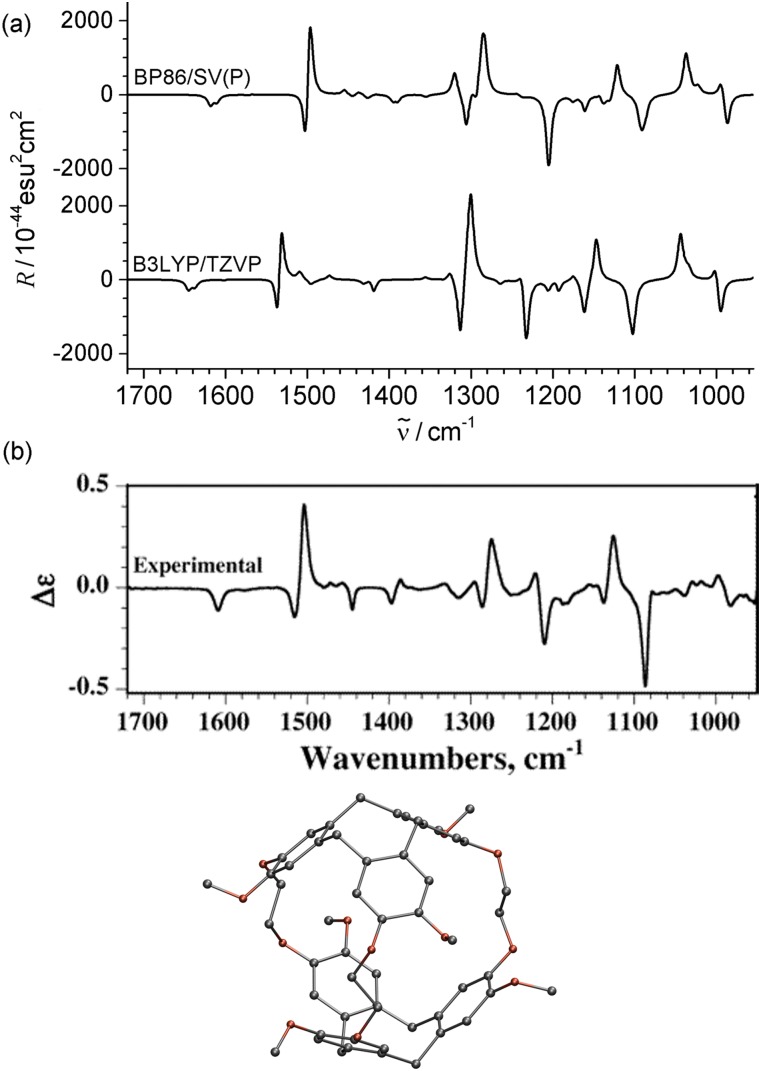
(a) Simulated VCD spectra for different combinations of basis sets and functionals. (b) Experimental VCD spectrum and an image of cryptophane-A. Reprinted with permission from Reiter *et al.*, J. Chem. Phys. **146**, 054102 (2017). Copyright 2017 AIP Publishing LLC.

The calculations of icosahedral carbon clusters and organometallic compounds in Ref. 222 reproduce the measured spectra very well. Herein, we only discuss the spectra of cryptophane-A shown in Fig. 12. The differences between the calculated spectra are smaller than those to the experimental spectrum, where the much more cost-effective combination of BP86^235,236^/def2-SV(P)^117^ needs only 2% of the time of the combination B3LYP^215,216^/def2-TZVP.^117^ Nevertheless, the calculation of VCD spectra for mid-sized molecules of ∼100 atoms at the hybrid DFT and triple-*ζ* basis set level is feasible within a few days (for an Intel Xeon E5-2687W v2 CPU @ 3.4 GHz.)

### I. CVS-ADC(2) and CVS-CC2 for core spectroscopy

Core-excited states are located above the ionization limit of the valence electrons and are embedded in a continuum of high-lying ionized and doubly excited states. They are not discrete eigenfunctions of the Hamiltonian but resonances that are not readily accessible by the usual techniques for bound states. The core–valence separation (CVS) approximation^237^ decouples the core excited states from the valence states by skipping in the second-quantized Hamiltonian the coupling terms that change the number of electrons in core orbitals.

For valence states and transition moments between valence states, the CVS approximation is equivalent to the frozen-core approximation. The excitation energies and amplitudes for singly core excited states are obtained as eigenpairs of the block of the Jacobian matrix **A** or, for ADC(2), the secular matrix with one core hole index (1*ch*),

$$\left(\begin{array}{cc} A_{val,val}\; & 0\\ 0\; & A_{1ch,1ch} \end{array}\right)\left(\begin{array}{c} 0\\ E_{1ch}^{f} \end{array}\right)=\omega_{1ch}^{f}\left(\begin{array}{c} 0\\ E_{1ch}^{f} \end{array}\right).$$ (1)

In addition to excitation energies, the CVS approximation has been implemented for transition strengths between core-excited states and the ground state or another core- or valence-excited state.^238^ For the transition moments, the neglect of the coupling terms between core-excited and valence states in the Hamiltonian leads to simplifications that reduce computational costs, as some of the Lagrange multipliers that appear in the expressions for transition moments vanish for excitations between valence and core-excited states (see Table VII).

**TABLE VII.** Timings for a CVS-CC2/aug-cc-pVTZ calculation (1334 functions) for core excitations in pentacene, C_22_H_14_, *D*_2*h*_ symmetry, using the 16 cores of two Xeon E5-2609 v4 CPUs and 48 Gb RAM. ${\bar{M}}^{f}$ and ${\bar{N}}^{fi}$ are Lagrange multipliers needed for transition moments for ground-to-excited and excited-to-excited state transitions (cf. Ref. 239), respectively.

Equations	Core holes	Wall time (s/iter.)
Ground state equations	0	24
Eigenvalue equations	0	18
Eigenvalue equations	1	23
Equations for ${\bar{M}}^{f}$	0	14
Equations for ${\bar{M}}^{f}$	1	(Vanishes)
Equations for ${\bar{N}}^{fi}$	0 → 0 or 1 → 1	22
Equations for ${\bar{N}}^{fi}$	0 → 1	(Vanishes)
Equations for ${\bar{N}}^{fi}$	1 → 0	24

**FIG. 13.**
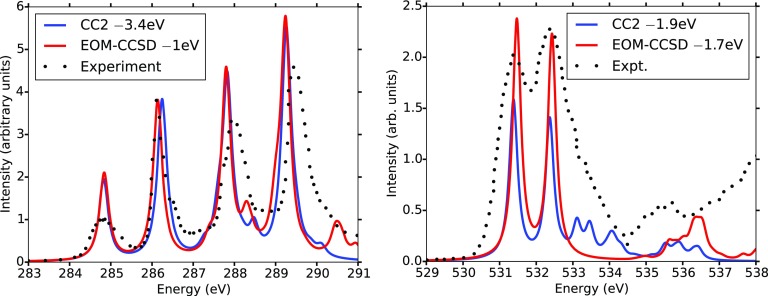
Comparison of the fc-CVS-CC2 and fc-CVS-EOM-CCSD NEXAFS spectra at the carbon and oxygen K-edges in uracil. Basis set 6-311++G^**^.^246,247^ The CCSD results are from Ref. 244. Experimental spectra have been re-digitized from Ref. 245.

To date, the accuracy of CC2 for core excitations has been scarcely investigated^240–242^ and almost only for a variant where the CVS is applied during the determination of the target states.^243^ The evidence collected from those studies indicates that CC2 yields reasonably accurate results for 1*s* → π^*^ transitions at the carbon K-edge. The accuracy tends to deteriorate when addressing more energetic edges and/or core transitions of Rydberg character. This behavior can be partly ascribed to the increased importance of double excitations, which are lacking at the CC2 level, to describe relaxation effects at the more energetic K-edges. For the CVS-CC2 variant implemented in TURBOMOLE,^238^ we show in Fig. 13 a comparison of the near edge X-ray absorption fine structure (NEXAFS) spectra of uracil at the carbon and oxygen K-edges with the fc-CVS-EOM-CCSD results of Ref. 244 and with experiment.^245^ Rigid shifts as indicated in the legends have been applied to roughly align with the experimental peak. Besides the different shifts required for the two methods, the main differences in the spectra are observed at the oxygen K-edge moving toward the region of Rydberg excitations.

The core-level spectra of ground and excited states can be computed with the ricc2 program at the ADC(2) and the CC2 levels of theory for singlet and triplet states.^22,238,248^

### J. Ionized, electron-attached and excited states: The *GW*–BSE method

**FIG. 14.**
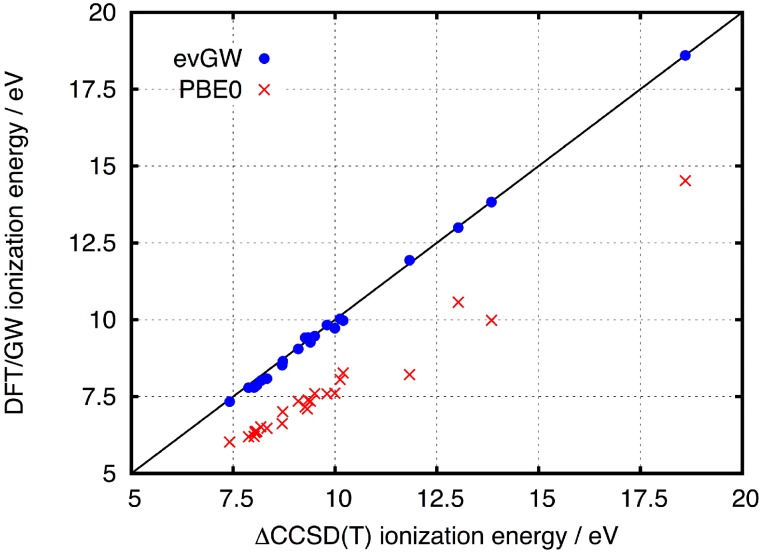
Comparison of ev*GW* QP energies and PBE0/def2-SVP orbital energies to the ionization potentials obtained from CCSD(T)/cc-pVDZ^249^ for a test set of 24 ionization potentials.

The *GW* method has developed into an established tool for calculating quasiparticle (QP) energies of various systems.^250^ Since its first implementation in TURBOMOLE,^251–253^ recent developments have focused on improving its applicability to extended molecular systems^33^ and to heavy-element chemistry using TURBOMOLE’s two-component framework.^33^ The widely used perturbative *G*_0_*W*_0_ method aims at a “one-shot” improvement of Kohn–Sham orbital energies via the self-energy Σ_C_,

$$\varepsilon_{p}^{\left(i+1\right)}=\varepsilon_{p}^{\left(0\right)}+\left\langle \phi_{p}|\Sigma_{C}\left(\varepsilon_{p}^{\left(i\right)}\right)+\Sigma_{X}-v_{XC}|\phi_{p}\right\rangle ,$$ (2)

where Σ_*X*_ is the Hartree–Fock exchange and *v*_XC_ is the exchange–correlation potential. The $\varepsilon_{p}^{\left(0\right)}$ are the Kohn–Sham orbital energies, and in the *G*_0_*W*_0_ method, only one single, straight iteration is performed to generate the quasiparticle energies $\varepsilon_{p}^{\left(1\right)}$. When this one iteration is performed in the Newton–Raphson manner, the method is denoted “linearized *G*_0_*W*_0_.” Quasiparticle energies obtained via Eq. (2) are direct approximations to the ionization potential (IP) of an electron in the corresponding orbital, with the IP simply being the negative QP energy. Self-consistent schemes going beyond the *G*_0_*W*_0_ approximation have been implemented in TURBOMOLE, targeting either self-consistency in the QP energies (termed eigenvalue self-consistent *GW*, ev*GW*) or full self-consistency of the QP equation (qs*GW*).^254^ A starting point dependence is still observed for the computationally cheaper ev*GW*. As shown in Fig. 14, the ev*GW* QP energies vastly improve the Kohn–Sham orbital energies toward approaching the corresponding ionization energy, reducing the mean absolute error from 2.2 eV to 0.1 eV. Since a *G*_0_*W*_0_ or ev*GW* calculation in TURBOMOLE usually takes less time than the corresponding (hybrid) DFT calculation and removes the hassle of dealing with the charged systems, it is often easier to use than ΔSCF approaches. This is especially true when non-valence states are targeted, which is trivial with *GW* but not with ΔSCF.

**FIG. 15.**
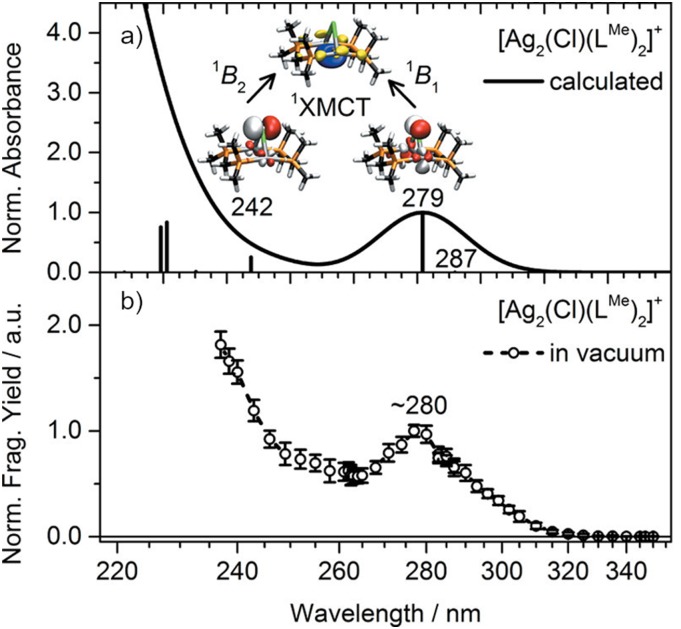
Comparison of the simulated (PBE0-D3(BJ)/ev*GW*-BSE/def2-SVPD) (a) and measured (b) UV photodissociation spectra of the investigated [Ag_2_(Cl)(dmpm)_2_]^+^ (dmpm = L^Me^) complex. Reprinted with permission from Kruppa *et al.*, J. Phys. Chem. Lett. **9**, 804–810 (2018). Copyright 2018 American Chemical Society.

The Bethe–Salpeter equation has evolved into a useful tool for the theoretical description of electronically excited states, as shown by various benchmark studies.^250,254–258^ Starting from *GW* QP energies, it involves exactly the same time and cost per iteration as TDDFT within the RI-*K* approximation.^259^ The *GW*-BSE method therefore has become an efficient tool to deal with extended molecular systems, which could previously only be tackled using TDDFT. In contrast to most density functional approximations, the *GW*-BSE method features a correct asymptotic behavior and is therefore able to describe charge-transfer excitations with relative ease. The *GW*-BSE implementation in TURBOMOLE has recently been extended to include relativistic effects based on a two-component formalism and is to date also unique in the possibility to perform correlation-kernel-augmented BSE (termed cBSE) calculations.^33^ cBSE is able to describe singlet, triplet, and charge-transfer excited states accurately on the same footing and has been shown to yield improved results for various organic molecules^260^ as well as for complexes containing metal centers, especially when relativistic effects are included.^33^ cBSE is therefore an interesting alternative if a correct description of singlet–triplet gaps is important, for example, in molecules that feature thermally activated delayed fluorescence (TADF). Investigations on the photodissocation process of an [Ag_2_(Cl)(dmpm)_2_]^+^ complex [dmpm = bis(dimethylphosphino)methane], presented in Fig. 15, also exhibit excellent agreement between experimentally obtained data and simulations.^261^

### K. Valence and core ionization potentials from GKS-spRPA

**FIG. 16.**
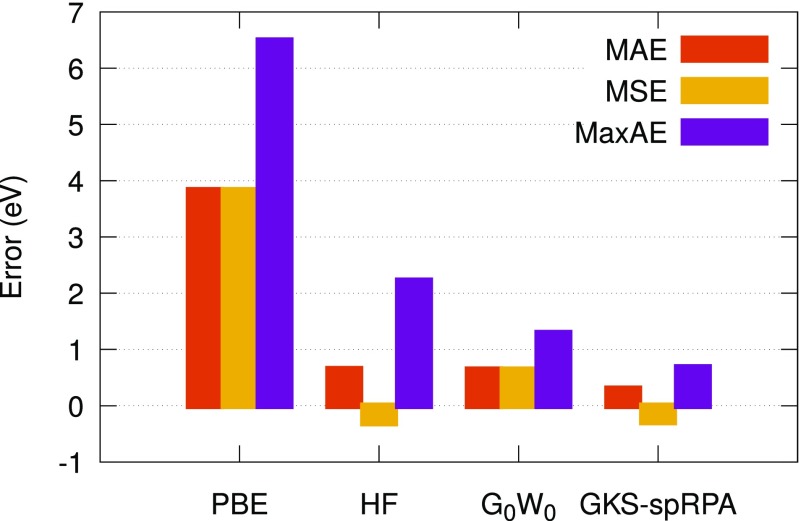
Mean absolute (MAE), mean signed (MSE), and maximum absolute (MaxAE) errors (eV) for the highest occupied molecular orbital energies for the GW27 test set. The reference method is CCSD(T), and the def2-TZVPP basis set was used for all the methods. PBE potential was used for the *G*_0_*W*_0_ and GKS-spRPA calculations.

A generalized Kohn–Sham semicanonical projected random-phase approximation (GKS-spRPA)^262^ method has been implemented within the rirpa module. In GKS-spRPA, the spRPA energy is variationally minimized with respect to the density matrix, D, under the constraints of orbital orthonormality and *N*-representability, leading to an effective one-particle equation

$$H_{0}^{\text{sp}-\text{RPA}}\left[D\right]\phi_{p}=\varepsilon_{p}\phi_{p}\text{,}$$ (3)

which is solved self-consistently. However, the GKS-spRPA results still show a minor dependence on the underlying potential of the density functional approximation. At the stationary point, in addition to obtaining a variational total energy, the eigenvalues of the effective potential, $H_{0}^{\text{sp}-\text{RPA}}$, yield the one-particle orbital energies, *ε*_*p*_, which are approximate IPs. GKS-spRPA IPs account for the static Hartree-exchange effects, orbital correlation (OC), orbital relaxation (OR), and static changes to the density due to correlation effects (C,s). For valence IPs of neutral molecules in the GW27 benchmark test set,^251,263^ GKS-spRPA reduces the errors by ∼50% compared to the *G*_0_*W*_0_ method (see Fig. 16).

**FIG. 17.**
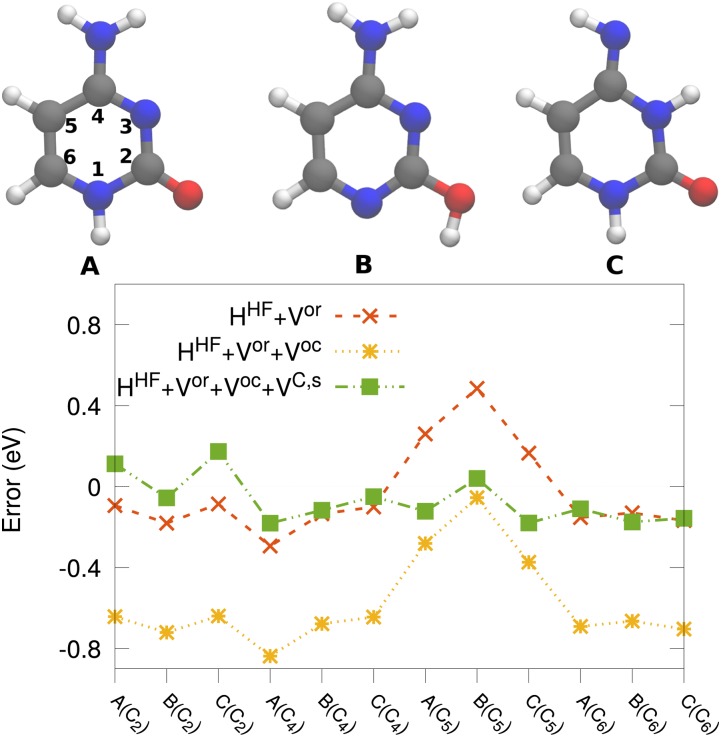
(Top) Tautomers of cytosine considered in this study. The following color scheme was used for the atoms: H (white), C (gray), N (blue), and O (red). (Bottom) Errors in *d*-GKS-spRPA based C(1*s*) CEBEs for cytosine tautomers compared to CVS-ADC(4) values.^264^ HF exchange is denoted as HF. For the *d*-GKS-spRPA calculations, def2-TZVPP basis sets and the PBE potential (grid size m5) were used. The molecular geometries were optimized using the PBE energy functional. Reprinted with permission from Voora *et al.*, J. Chem. Phys. **151**, 134106 (2019). Copyright 2019 AIP Publishing LLC.

Compared to modeling valence ionization, the simulation of core-ionization poses special challenges to theoretical methods due to large orbital relaxation effects, high ionization energies, and strong relativistic effects for heavy nuclei. Since the one-electron Hamiltonian from GKS-spRPA incorporates correlation and relaxation effects in a balanced fashion, both valence and core ionization energies can be modeled without any tuning parameters. For example, GKS-spRPA can help simulate the complex core-ionization spectra of cytosine. Cytosine, at ∼450 K, exists as three tautomers—A, B, and C (Fig. 17)—as demonstrated in a combined experimental and theoretical study.^264^ The study showed that the variations in the position of the proton in these three tautomers leads to only six resolved-features in the C(1*s*) X-ray photoelectron spectroscopy (XPS). CVS-ADC(4) was shown to provide reliable estimates for the core electron binding energies (CEBEs) for this case. For all three tautomers, we find that the *d*-GKS-spRPA (i.e., GKS-spRPA Hamiltonian within a diagonal approximation) based C(1*s*) CEBEs are within 0.2 eV of CVS-ADC(4) values.^265^ Since the *d*-GKS-spRPA approach is about two orders of magnitude computationally cheaper than CVS-ADC(4) for core ionization, it emerges as an appealing method for studying core IPs. We note that all four components of the spRPA potential are necessary for obtaining the least errors.

GKS-spRPA and *GW* methods, besides ΔSCF approaches, thus constitute the main approaches for computing IPs in TURBOMOLE. The (*d*-)GKS-spRPA, *G*_0_*W*_0_, and ev*GW* methods have similar scaling with different prefactors, resulting in the following order of computational effort: ΔSCF < *G*_0_*W*_0_ < ev*GW* < *d*-GKS-spRPA < GKS-spRPA < qs*GW*. GKS-spRPA methods provide IPs and total energies, while *GW* methods provide IPs only. *GW* methods can yield photoelectron intensities, which are missing from GKS-spRPA. For reliable estimates of core IPs and their chemical shifts, we recommend the use of *d*-GKS-spRPA and qs*GW* methods.^265,266^

### L. Local coupled-cluster excitation energies of large chromophores

The program pnoccsd, introduced with release version 7.0, provides low-scaling implementations of a variety of coupled-cluster models based on the pair natural orbital approximation. Recently, its functionality has been extended^7,8,267,268^ to the computation of excitation energies with the local models PNO-CIS(D), PNO-CIS(D_*∞*_), PNO-ADC(2), PNO-CC2, PNO-ADC(2)-x, and PNO-CCSD.

The basis for the implementation of excitation energies for these methods is the state-specific PNO ansatz introduced by Helmich and Hättig.^7^ Within this ansatz, a separate PNO basis is constructed for each excited state *n* according to

$$\underset{ab}{\sum }d_{aā}^{ij}D_{ab}^{ij}\left(\omega ,R_{1}\right)d_{b\bar{b}}^{ij}=\delta_{ā\bar{b}}n_{ā}^{ij},$$ (4)

where $d_{aā}^{ij}$ are the PNO expansion coefficients and $n_{ā}^{ij}$ are the natural occupation numbers. For each state, the PNO expansion is truncated individually according to a user-defined threshold *T*_PNO_, and hence, all PNOs with $n_{ā}^{ij}<T_{PNO}$ are discarded. The approximate CIS(D)-like density $D_{ab}^{ij}$ from which the PNOs are constructed introduces the nature of the excitation process through the excitation energy *ω* and the excited-state singles amplitudes *R*_1_. To account for possible changes in the physical character of excited states (state switches) during the optimization, the excited-state eigenvalue problem is solved self-consistently, i.e., a new PNO basis is constructed whenever *ω* or *R*_1_ changed significantly.

**FIG. 18.**
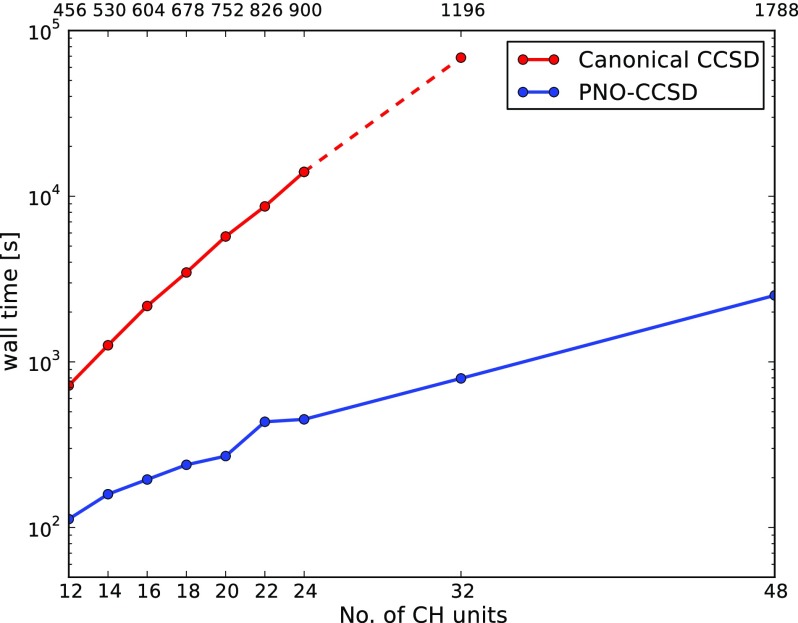
Wall time of a single Jacobian right transformation for canonical and PNO-CCSD for alkene chains with the def2-TZVP basis set. For the PNO-CCSD calculations, *T*_PNO_ = 10^−7^ was chosen. The dashed line for canonical CCSD indicates that these timings were obtained by an extrapolation since the actual calculation was not possible due to the steep scaling of computational resources.^268^ Reprinted (adapted) with permission from Frank and Hättig, J. Chem. Phys. **148**, 134102 (2018). Copyright 2018 AIP Publishing LLC.

The local approximations reduce the scaling of the computational costs with the system size, e.g., for PNO-CCSD from $O\left(N^{6}\right)$ to an at most cubic scaling (cf. Fig. 18), and make these methods applicable to much larger systems.

**FIG. 19.**
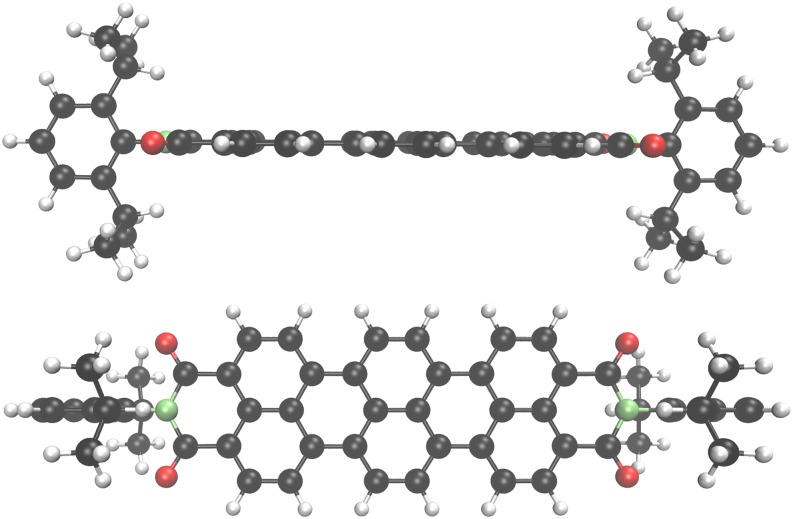
Side and top views on the rylendiimide *N*,*N′*-(2,6-diisopropylphenyl)-terrylene-3,4:11,12-tetra-carboxidiimide optimized at the COSMO-B3LYP/def2-TZVP (*ε*_r_ = 4.89, *n*_D_ = 1.446) level of theory. Color code: black, carbon; red, oxygen; and green, nitrogen.

Recently, we have combined PNO-CC2 with COSMO and polarizable embedding (PE) to include environmental effects. For the first transition of the rylenediimide shown in Fig. 19, the COSMO-PNO-CC2/aug-cc-pVDZ approach predicts a vertical excitation energy of 1.87 eV in chloroform, red-shifted by 0.22 eV^269^ compared to the isolated molecule. The computed excitation energy is in strikingly good agreement with the experimental result of 1.91 eV.^270^ For this example with 1886 atomic orbitals, the transformation of the Jacobi matrix with a trial vector from the right takes about 323 s using 40 threads on two Intel Xeon Gold 6230 CPUs. The evaluation of the COSMO contributions amounts to roughly 10% of the total timings.

### M. Beyond UV/vis: CC2 for nonlinear and induced spectra

During the last few years, the ricc2 program has been extended to the calculation of non-linear and induced spectra at the RI-CC2 level using the coupled-cluster response theory approach.^239,271^ This comprises two-photon absorption,^272^ two-photon circular dichroism,^273^ and magnetic circular dichroism^274^ spectra as well as transition moments from singlet ground and to excited triplet states induced by spin–orbit coupling to describe ordinary^275^ and circularly polarized phosphorescence.^276^ All these properties have in common that they additionally require the first-order response amplitudes and perturbed density matrices. To evaluate them without giving up ricc2’s design paradigm^22^ of avoiding the storage of doubles amplitude vectors, which would hinder the application to large molecules, the RI approximation is combined with a numerical Laplace transform of the orbital energy denominators^34^ for the unperturbed ground or excited-state amplitudes.

**FIG. 20.**
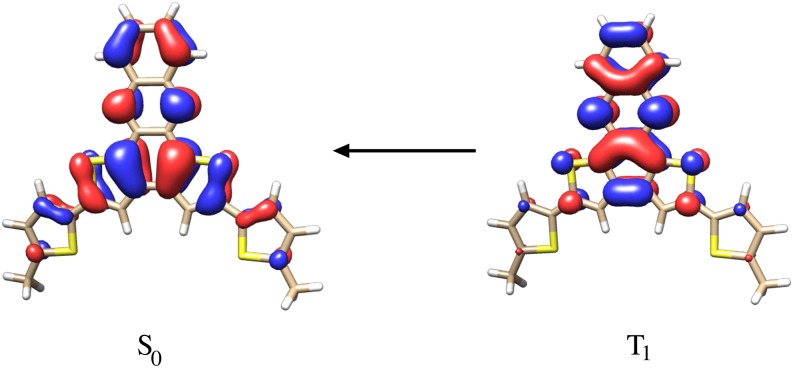
Dominant natural transition orbital pair for the T_1_ → S_0_ transition in a metal-free phosphorescent emitter.^275^ Reprinted (adapted) with permission from Helmich-Paris *et al.*, J. Chem. Theory Comput. **12**, 1892–1904 (2016). Copyright (2016) American Chemical Society.

The spin–orbit induced T_1_ → S_0_ transition strength of a metal-free phosphorescent emitter^277^ (Fig. 20) was, for example, computed with RI-CC2 using the aug-cc-pVTZ basis set, the spin-free X2C Hamiltonian, and a mean-field spin–orbit operator.^275^ For the calculation, 1856 orbital and 4292 auxiliary basis functions were used. Point group symmetry (*C*_2*v*_) was also exploited for all time-critical intermediates. The CC2 calculation ran in parallel on eight cores and took 7 days and 3 h on two Intel Xeon Harpertown (E5430) CPUs. For the calculation, we used the T_1_ excited-state equilibrium structure, and the T_1_ → S_0_ transition energy (1.80 eV) agrees very well with experiment (1.83 eV).^277^ However, the SOC-PT-CC2 high-temperature average of the phosphorescence lifetime is clearly overestimated (1.3 × 10^5^ ms) when compared with the experimental lifetime^277^ (1.1 × 10^1^ ms). Most likely, this is caused by omitting vibrational effects, which become important in the long-lifetime regime.

Alternatively, spin–orbit induced transition strengths can be computed with the ricc2 program by using the SO-X2C Hamiltonian in CC2 calculations.^111^ Although being more accurate because spin–orbit coupling is accounted for variationally at the HF and CC2 level, the computational costs of SO-X2C-CC2 calculations are much larger than with non-relativistic CC2 due to complex quantities and algebra. The SOC-PT approach only works with real quantities and is roughly seven times faster than SO-X2C-CC2, even though five times more response equations and seven times more one-electron density matrices need to be computed.^275^

### N. Nonlinear optical properties of large molecules from TDDFT

Several nonlinear optical properties of molecules, such as two-photon absorption (TPA) cross sections, second-harmonic generation (SHG) amplitudes, and excited-state absorption (ESA) oscillator strengths, are accessible in the framework of quadratic response theory. These properties provide sensitive probes of molecular structure and are instrumental in the development of novel functional materials. Quadratic response functions contain a multitude of information, with the SHG amplitudes being obtainable from the dynamic hyperpolarizability tensor ***β***(*ω*, *ω*), while TPA cross sections and ESA intensities result from the first- and second-order residues of the quadratic response at electronic excitation energies, respectively. The implementation of TDDFT quadratic response^278^ is based on the density-matrix formalism of TDDFT^65^ and takes advantage of molecular point group and permutational symmetry. These techniques enable calculations of nonlinear optical properties in molecules with hundreds of atoms and thousands of basis functions on a single computer node.

**FIG. 21.**
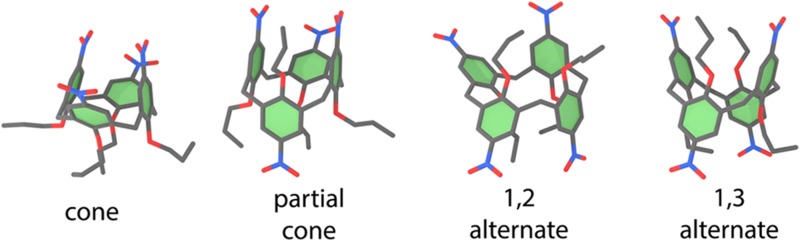
Schematic structures of nitrocalix[4]arene conformers with large experimental SHG intensities. Reprinted with permission from Parker *et al.*, J. Chem. Theory Comput. **14**, 807–819 (2018). Copyright 2018 American Chemical Society.

**FIG. 22.**
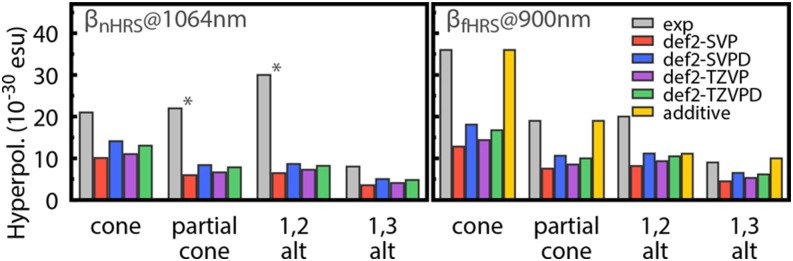
Computed dynamic hyperpolarizabilities of nitrocalix[4]arenes of Fig. 21 with the PBE0 functional^279^ at 1064 nm and 900 nm in comparison with the experiment and an additive scheme. Reprinted with permission from Parker *et al.*, J. Chem. Theory Comput. **14**, 807–819 (2018). Copyright 2018 American Chemical Society.

Using this implementation, the nonlinear optical behavior of conformers of nitrocalix[4]arenes (Fig. 21) were studied. These molecules contain the D–*π*–A structural pattern in which an electron-donating group (D) and an electron-accepting group (A) are connected via a conjugated *π* system and have either dipolar or approximate octupolar symmetry. Because of their large experimental second-order responses at low excitation energies, nitrocalix[4]arenes are interesting prototypes for molecular materials for SHG. The computed dynamic hyperpolarizabilities of nitrocalix[4]arenes at 1064 nm and 900 nm are shown in Fig. 22. The experimentally observed ordering of conformers by increasing hyperpolarizability is well reproduced. On the other hand, computed hyperpolarizabilities, like other response properties, are sensitive to the choice of basis sets. Using basis sets with diffuse augmentation^280^ generally improves the accuracy of the predictions. In contrast to linear polarizabilities, the basis set convergence is not monotonic.

**FIG. 23.**
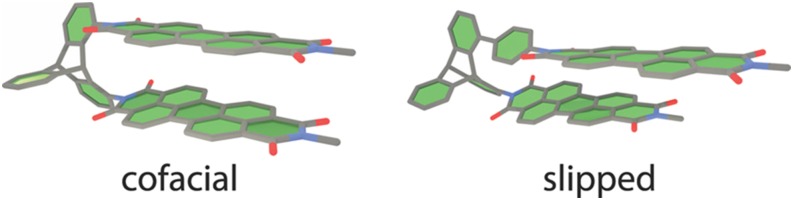
Structures of facial and slipped-stacked PDI dimers. Reprinted with permission from Parker *et al.*, J. Chem. Theory Comput. **14**, 807–819 (2018). Copyright 2018 American Chemical Society.

**FIG. 24.**
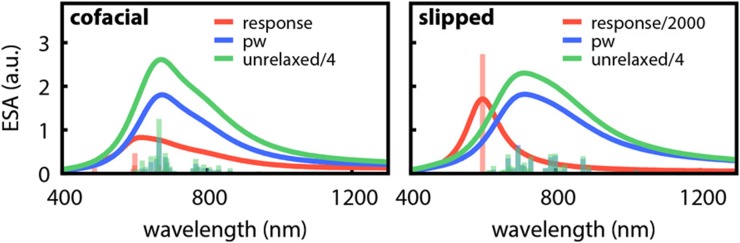
Computed excited-state absorption spectra in PDI dimers with the PBE0 functional with different approaches to the orbital response.^279^ Stick spectra indicate the cross sections of the state-to-state transitions. Note that the uncorrected results of the response calculation are scaled by 1/2000. Reprinted with permission from Parker *et al.*, J. Chem. Theory Comput. **14**, 807–819 (2018). Copyright 2018 American Chemical Society.

A vexing issue in quadratic response calculations is that they exhibit spurious poles, which lead to unphysical divergences in transition properties between states *M* and *N*, whenever their energy difference matches the excitation energy from the ground state to another excited state *K*, |*E*_*M*_ − *E*_*N*_| ≈ *E*_*K*_ − *E*_0_. This is a fundamental problem of the response formalism^281^ but is exacerbated in computations involving large systems with dense excitation spectra. An example is the ESA spectra of perylene diimide (PDI) dimers, which are model systems for energy transfer in artificial light harvesting systems (Fig. 23). The effect of the divergences in the ESA spectra is obvious in the slipped-stacked conformation (Fig. 24, right panel) in which a single divergent transition dominates the ESA spectral shape. Two approaches have been developed to prevent these unphysical divergences: In the pseudowavefunction (PW) approach, the orbital relaxation contributions to the quadratic response are treated statically, whereas in the unrelaxed approach, all orbital relaxation is neglected. While the unrelaxed approach generally overestimates the transition properties, the PW approach offers a reasonable trade-off between an accurate description of orbital relaxation and numerical stability, as illustrated in Fig. 24 (left panel).

### O. Improved treatment of triplet excitations using local hybrid functionals

**FIG. 25.**
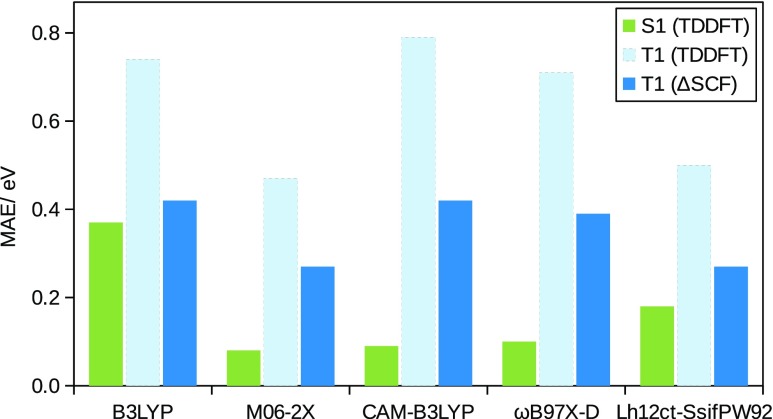
Mean absolute errors (MAEs) with respect to the CC2/CBS reference of a subset of functionals and data from Ref. 289 regarding S_1_ and T_1_ TDDFT excitation energies of captodatively stabilized biradicaloids. Results for the T_1_ states obtained with the ΔSCF approach are also shown.

The good performance of local hybrids (cf. Sec. III C) for ground-state properties^166,282^ motivated the implementation of linear-response TDDFT for these functionals, which was realized in the escf module.^283^ In an early benchmark study of vertical excitation energies,^284^ remarkable performance was seen for several challenging types of excitations such as Rydberg and core excitations, which reflects the variable EXX admixture that ranges from high values in the core and asymptotic regions to lower values in the valence region. Most promising perhaps was the outstanding performance for valence triplet excitations with a mean absolute error of 0.16 eV for the Thiel test set^285^ (B3LYP: 0.45 eV and M06-2X:^286^ 0.23 eV).^284^ Triplet excited states are notoriously difficult for TDDFT^287^ but of prime importance in several applications. Here, we focus on research in the field of singlet fission,^288^ where a high-throughput screening for chromophores meeting the energy criterion $E\left({\text{S}}_{1}\right)-E\left({\text{S}}_{0}\right)\approx 2\left[E\left({\text{T}}_{1}\right)-E\left({\text{S}}_{0}\right)\right]$ could be used to find candidates worthwhile for further investigation regarding their applicability in dye-sensitized solar cells. In a benchmark study^289^ of prototypical captodatively stabilized biradicaloids,^290^ a reliable protocol employing the local hybrid TDDFT implementation was suggested. The T_1_ states of these molecules are suspected to exhibit appreciable static correlation that leads to rather large errors with standard functionals. B3LYP, for instance, gives a large MAE of 0.74 eV for the T_1_ states within the (full linear response) TDDFT approach and a considerable error of 0.42 eV when the ΔSCF scheme is used (cf. Fig. 25). Range-separated hybrids such as CAM-B3LYP^217^ and *ω*B97X-D^169^ were found to be reliable for the S_1_ states but show the same pitfalls for the T_1_ excitations. The simple first-generation LSDA-based Lh12ct-SsifPW92^291^ was found to be the most successful local hybrid, improving substantially on the T_1_ results, both for the TDDFT and ΔSCF approach. Apparently, the implicit simulation of left–right correlation by local hybrids^165^ is key to their success for these demanding molecules. The M06-2X functional was found to be on par with the local hybrids for the T_1_ excitations but uses a considerable amount of empirical parameters, whereas only two are used in Lh12ct-SsifPW92. The MAEs for the T_1_ excitations are further reduced and brought close to the target accuracy of 0.2 eV when favorable error compensation with the underlying DFT structure optimization is exploited.^289^ We note that the suggested protocol can be tailored to a given project’s needs, as done for a recent application to characterize a new singlet-fission chromophore.^292^

As previously described for ground states (cf. Sec. III C), the balance between reduced delocalization errors and simulation of left–right correlation in local hybrids makes them particularly useful to treat the MV system. As seen in another application of the TDDFT implementation of local hybrids to the intervalence charge-transfer bands in dinuclear MV transition-metal complexes, this also seems to apply to excited states.^293,294^

To extend the applicability of local hybrids in the field of photochemistry, their excited-state gradients were recently implemented in the egrad module.^295^ First assessments revealed competitive performance for excited-state structural parameters and vibrational frequencies as well as excellent performance for adiabatic triplet excitation energies.^295^

### P. Vibronic spectra of polycyclic aromatic hydrocarbons

**FIG. 26.**
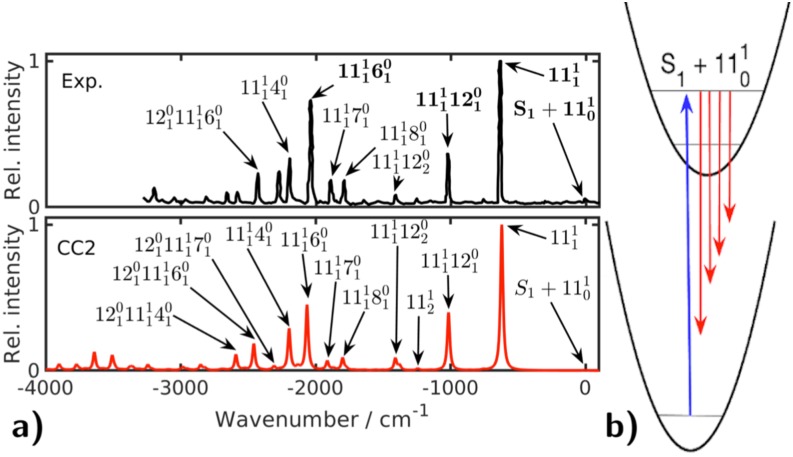
(a) Single vibronic level (SVL) fluorescence spectrum of anthracene excited to the S_1_+11 vibronic level (black: experimental,^296^ red: calculated using the radless module). (b) Schematic representation of the SVL process; upon excitation to the S_1_+11 level (blue arrow), emission to the various vibrational levels in the ground state occurs (red arrows). Reprinted (adapted) with permission from Tapavicza, J. Phys. Chem. Lett. **10**, 6003–6009 (2019). Copyright 2019 American Chemical Society.

Vibrationally resolved electronic absorption and emission spectroscopy are of fundamental importance in molecular physics^296^ because they provide a powerful tool to study molecular processes such as internal conversion or intersystem crossing.^297,298^ The prediction of electronic absorption bands is often done based on an ensemble of structures obtained from *ab initio* or classical molecular dynamics; this approach leads to accurate absorption spectra if the cause of broadening is due to the presence of several conformers.^299,300^ However, since in molecular dynamics, nuclear degrees of freedom are described by classical physics, the effects of the nuclear quantum nature cannot be captured accurately.^301^ To include the quantum nature of nuclear vibrations, the prediction of vibrationally resolved electronic spectra, in general, follows two routes:^302^ prediction in the frequency domain^303^ and prediction in the time domain.^304^ Time-independent (TI) approaches formulated in the frequency domain require the computation of Franck–Condon (FC) factors of the vibronic transitions. This becomes computationally expensive with an increase in the number of vibrational degrees of freedom. For large systems,^305,306^ time-domain approaches are computationally more efficient than TI approaches. The radless module provides a time-dependent implementation to compute vibrationally resolved absorption and emission spectra. The method is implemented in the zero temperature limit, which is the most important case, because typically vibrational modes that require quantum treatment are only occupied in its lowest vibrational state at ambient temperatures. Compared to the temperature-dependent approaches, besides its simplicity, the zero temperature approach exhibits better numerical stability at low temperatures.^302^ A new extension of radless furthermore allows one to compute emission and absorption spectra that arise from a singly occupied vibrationally excited initial states, allowing to simulate single vibronic level (SVL) fluorescence^307^ (Fig. 26) and vibrationally promoted electronic resonance (VIPER) spectra.^308^

### Q. Radiationless decay pathways with IRCs and ADC(2)

**FIG. 27.**
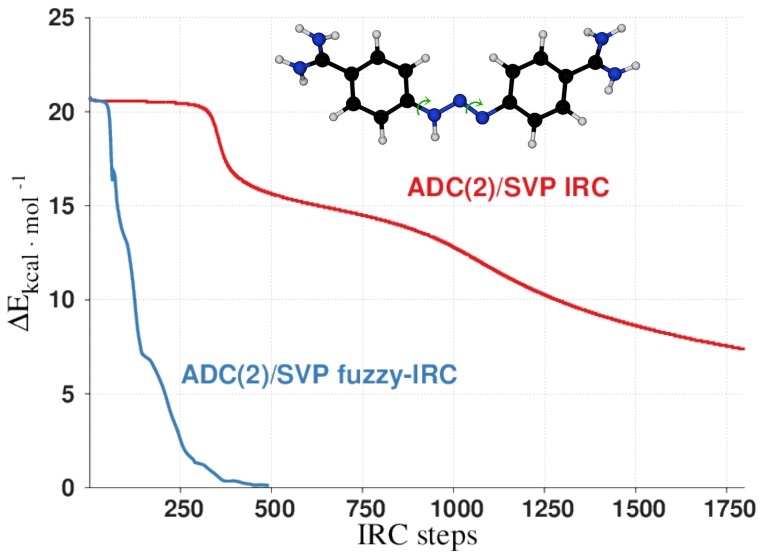
Comparison between the IRC and fuzzy-IRC algorithms in the calculation of the S_1_ excited-state radiationless decay bicycle-pedal decay pathway of berenil. The energy of the S_1_ minimum has been set to zero. For comparison, the step size of 80 hartree unit of time has been used in both calculations.

The time-evolution of molecular systems in electronic excited states can be studied by means of nonadiabatic mixed quantum–classical dynamics without the need to precompute the potential energy surfaces. In particular, the wavefunction-based ADC(2) method found applications for the excited-state simulations of the heterocyclic systems and small super-molecular clusters.^309^ Unfortunately, for flexible systems with more than ∼30 atoms, *ab initio* molecular dynamics calculations beyond the DFT level are computationally expensive. Thus, it is difficult for flexible systems to obtain a statistically converged ensemble of trajectories at this level, restricting the application of these techniques. However, to obtain a qualitative picture of the photochemical and radiationless decay pathways, it is often sufficient to study only the thermodynamically averaged reaction paths. The intrinsic reaction coordinate (IRC) analysis^310^ has an established application for studying the connectivity between potential energy surface minima and transition states. The DRC script can be combined with computational methods with the available excited-state gradients to study the radiationless decay pathways that are starting from the Frank–Condon point and can be used, similar to the ground state, to study the connectivities between transition states and minima on the electronic excited-state potential energy surfaces. For the berenil molecule (Fig. 27), the IRC calculations for the radiationless decay pathways of the excited states show that the excited-state relaxation is a two-phase process: A N=N bond elongation first occurs to approach an excited-state transition state and is followed by a volume-conserving bicycle-pedal motion to the S_1_ minimum. This mechanism is in agreement with time-resolved fluorescent up-conversion data.^311^ Moreover, the ricc2 module is capable of calculating transition moments between different excited states, which in combination with the IRC calculation of excited-state decay pathways has been used for berenil to interpret the time-resolved transient absorption spectra.^312^ Besides the conventional IRC calculations, a fuzzy acceleration technique has been implemented in the DRC script, which can reduce the computational cost of the IRC calculation significantly.^313^ In Fig. 27, the second phase of the S_1_ excited-state decay pathway of berenil has been computed with IRC and fuzzy-IRC methods. The IRC calculation gives a smooth energy profile; however, it is stuck in a floppy region of the potential energy surface and cannot converge to the minimum with a reasonable number of cycles. The smoothness of the fuzzy-IRC pathway can be controlled by adjusting the magnitude of increasing and decreasing the damping parameter and the time step of the (fuzzy-) IRC calculations. With these techniques, it is possible to study the photochemistry and photophysics of medium-sized chromophores and supermolecular systems routinely. Moreover, the evolution of excitation characters along the reaction pathways can be studied by means of natural transition orbital analysis, which has been implemented in the proper program.

### R. Fewest switches surface hopping implementation for unconstrained nonadiabatic molecular dynamics simulations

Tully’s fewest switches surface hopping (FSSH) algorithm provides an inexpensive semiclassical nonadiabatic molecular dynamics (NAMD) simulation method, where the effects of electronic transitions are captured by simulating an ensemble of independent trajectories of classical nuclei.^314^ Each trajectory propagates the classical nuclei on one of the adiabatic potential energy surfaces involved in the simulation and also carries with it an auxiliary reduced electronic density matrix that is used to choose the adiabatic potential energy surface to propagate the classical nuclei. All the observables and properties such as excited state lifetimes and reaction pathways are measured from the ensemble of trajectories. The required inputs for this semiclassical dynamics method are the energies and gradients of the electronic states along with the nonadiabatic coupling vectors between the states. Currently, analytical derivatives and coupling vectors^315^ are available only within the TDDFT framework, and some of its applications are highlighted in this section.

Of the five common nucleobases, thymine has the longest excited state lifetime, potentially making it more susceptible to excited state reactions.^316^ Having a dark S_1_ state and a close lying bright S_2_ state, the mechanism for radiationless decay is complicated and there is no consensus in the literature for the mechanisms and detailed decay pathways.^317–326^ Results from theoretical investigations vary greatly on the choice of electronic structure methods and semiclassical dynamics and are often at odds with known experimental evidence.^316,321,322,327–329^ TDDFT-based simulations of the photodeactivation of thymine were recently made possible with the implementation of state-to-state nonadiabatic couplings, which are extracted as residues of the pseudowavefunction approximation of the quadratic response (PW-QR) function.^278^ Parker *et al.* found that the semiclassical dynamics from the FSSH algorithm using the potential energy surfaces from linear response TDDFT and couplings from PW-QR TDDFT captures the excited dynamics of thymine, with remarkable agreement with experimental lifetimes.^330^ The total simulation time was 6.5 ps, which corresponded to an ensemble of 200 trajectories, each trajectory running on a single Intel Xeon E5-2680 v3 @ 2.50 GHz processor. The CPU time for each simulation time step was ∼240 s, making the energy consumption for each time step summed over the ensemble to be 1.6 kWh.

**FIG. 28.**
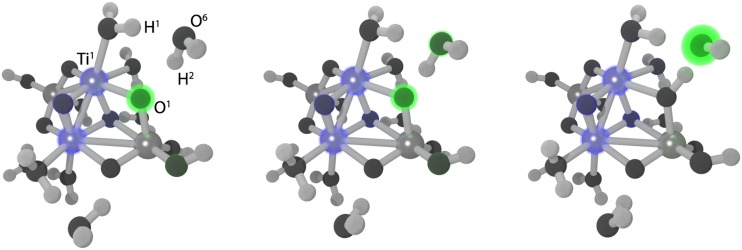
Snapshots from an NAMD trajectory at 200 fs (left), 213 fs (middle), and 218 fs (right) showing excited-state proton transfer for (TiO_2_)_4_(OH)_4_(H_2_O)_8_. Blue and green colors indicate negative and positive computed excitonic (electron–hole pair) charges, respectively. Reproduced from Muuronen *et al.*, Chem. Sci. **8**, 2179–2183 (2017). Published by The Royal Society of Chemistry. This article is licensed under a Creative Commons Attribution 3.0 Unported Licence, see https://creativecommons.org/licenses/by/3.0/ (link retrieved January 3, 2020).

Owing to its low cost and toxicity, and the hope of being directly usable in light harvesting, TiO_2_ nanoparticles have been one of the most popular model systems to study photoexcited exciton dynamics.^331–333^ Recent developments in computational tools have shed further light in the elusive mechanism of TiO_2_ nanoparticle water splitting.^334–336^ Muuronen *et al.* discovered a mechanism for the first step of the water oxidation reaction using the NAMD simulation feature.^337^ Using Tully’s FSSH algorithm as implemented in the module frog and the gradients and couplings of the electronic adiabatic states using the linear response TDDFT as implemented in egrad, the first unconstrained NAMD simulations on a small (TiO_2_)_4_(OH)_4_ cluster with additional 8–10 solvent water molecules were carried out. The study involved a total simulation time of 60 ps corresponding to an ensemble of 100 trajectories and revealed that the first step of water oxidation is a transfer of a hole from a bridging surface oxygen to the oxygen atom of a physisorbed water molecule via an excited-state proton transfer (see Fig. 28). Additional TURBOMOLE features such as calculation of natural bonding orbital (NBO) charges^338^ and excited-state density differences helped in the visualization of the charge transfer process. For each time step, the total CPU time was ∼65 min on Intel Xeon E5-2680 v3 @ 2.50 GHz processors, costing ∼13 kWh per step for the entire ensemble of trajectories.

Photochemical switches bear enormous potential as molecular logical gates, data storage, molecular motors,^339^ and pharmaceutical applications.^340^ The key quantity that describes the efficiency of a photochemical switch is the product quantum yield (PQY), which, in general, depends on the irradiation wavelength. Accurate prediction of the wavelength-dependent product quantum yield is thus an important goal of computational methods supporting the design and optimization of photoswitches. However, this is challenging because the PQY depends on several factors of which the major ones are theefficiency of absorption in a given frequency region, the raw branching ratio of the desired product, the presence of side reactions, and last but not least the fatigue, that is, the number of switching cycles that can be achieved before decomposition of the compound. Although, to some extent, predictions of trends of these quantities can be made based on the basis of static molecular properties,^341^ these quantities are, in general, highly dependent on the dynamics on the excited-state reaction and often on the equilibrium of ground-state conformers.^342^ A major factor that causes this dependency is the interplay between temperature effects and the topology of the manifold of potential energy surfaces for the ground and excited state. This makes the prediction of the PQY a difficult task if one wants to go beyond a static description. FSSH NAMD based on TDDFT^343–345^ allows users to obtain the raw branching ratios of photochemical reactions, which are often simply interpreted as the PQY.^346,347^ Going beyond raw branching ratios, the combination of FSSH and accurate methods to predict absorption spectra allow us to assess the wavelength dependency of conformationally controlled photochemical reactions. To obtain the wavelength dependency of a specific reaction pathway, the absorption spectra of all initial structures of the surface hopping trajectories (*σ*_tot_(*λ*)) and the subset of initial structures of trajectories that form a specific product (*σ*_P_(*λ*)) must be computed. Consistent with its definition,^348^ the PQY can then be calculated as the ratio of these two absorption spectra,

$$\Phi \left(\lambda \right)=\frac{\frac{1}{N_{\text{traj}}^{\text{P}}}\sum_{i}^{N_{\text{traj}}^{\text{P}}}\sigma_{i}\left(\lambda \right)}{\frac{1}{N_{\text{traj}}^{\text{tot}}}\sum_{i}^{N_{\text{traj}}^{\text{tot}}}\sigma_{i}\left(\lambda \right)}=\frac{\sigma_{\text{P}}\left(\lambda \right)}{\sigma_{\text{tot}}\left(\lambda \right)}.$$ (5)

**FIG. 29.**
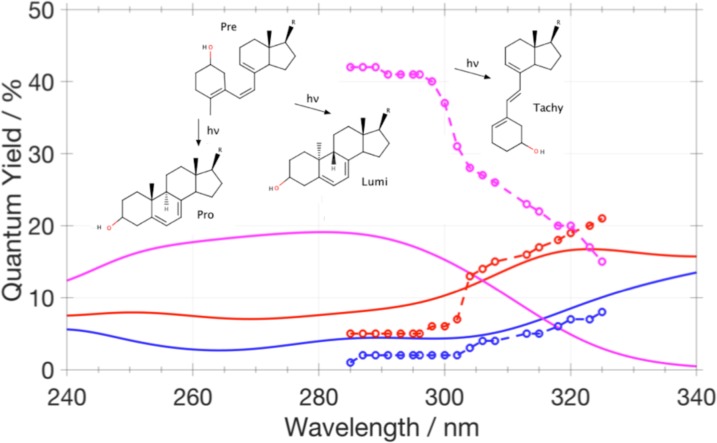
Calculated (solid)^349^ and experimental (circles)^350^ wavelength-dependent product quantum yields [magenta: tachysterol (Tachy); red: provitamin D (Pro); and blue: lumisterol (Lumi)]. Reprinted (adapted) with permission from Thompson and Tapavicza, J. Phys. Chem. Lett. **9**, 4758–4764 (2018). Copyright 2018 American Chemical Society.

Using 400 FSSH trajectories, this method was able to accurately predict the wavelength-dependent PQY of different photoproducts of previtamin D (Fig. 29) and substituted hexatriene derivatives.^349^

### S. COSMO

Solvation effects in TURBOMOLE can be treated with COSMO that has been described elsewhere in detail.^351–353^ The concept follows the ideas of dielectric continuum solvation models,^354^ namely, to embed the molecule in a dielectric continuum, build a cavity that includes the molecule and most of its electronic density, and compute the screening charges on the cavity surface. COSMO uses the boundary condition of a vanishing electrostatic potential on the surface and therefore treats the cavity as a metal-like surface.

The COSMO contribution to the Hamiltonian is a potential that depends on a scaling factor *f*(*ε*) with *ε* as finite permittivity, the screening charges of the cavity, and their positions. This potential can be used for Hartree–Fock and DFT calculations to obtain the energy and derivatives of the energy with respect to various variables, leading to properties including gradients, vibrational frequencies, NMR shieldings, and excited-state energies.

COSMO is applicable to homogeneously distributed non-polar solvents only. To overcome this limitation, the pairwise interaction of surface patches with screening charge densities is considered in statistical thermodynamics (COSMO-RS).^355–357^ The chemical potential that arises from COSMO-RS in addition to a term that takes care of combinatorial effects can again be used at the DFT level, resulting in the direct COSMO-RS (DCOSMO-RS) method.^358^ The COSMO-RS potentials of several solvents are available as parameters in TURBOMOLE, especially improving the solvation energy for polar solvents. DCOSMO-RS also strengthens the (virtual) hydrogen bonds between the solute and the solvent, which partly corrects the relative energy of conformations that have internal open or closed hydrogen bonds. The DCOSMO-RS implementation enables, among other things, optimization of structures of solutes with the additional terms of a COSMO-RS model present in the solvent potential. This is most significant for protic solvents, where hydrogen-bonding terms may be involved.

For post-Hartree–Fock methods such as MP2, different schemes can be applied to add the response of the solvent, the reaction field, to the MP2 energy and gradients.^359^ If applied to the Hartree–Fock step only, the MP2 correlation energy can be determined directly from the Hartree–Fock orbitals, which include the COSMO contributions. This is called the non-iterative energy-only scheme (PTE). If the MP2 density is used in an iterative manner also in the Hartree–Fock step to contribute to the screening charges, the reaction field is self-consistent (PTED). Both schemes have been implemented in TURBOMOLE for conventional MP2 and RI-MP2 calculations of energies and first-order properties. For the PTE scheme, gradients are available.

### T. SCRF schemes for excitation energies and spectra

Similar to the ground-state, excited-state energies can also be calculated for any method within the PTE scheme. As such calculations use the ground-state SCF reaction field potential throughout the calculation, they miss any effects from the response of the solvent on the electronic excitation in the solute. The unbalanced description of ground and excited states usually leads to blue-shifted excitation energies.

A more complete and balanced description for ground and excited states would be obtained by doing PTED calculations, where the reaction field potential and the density of the respective state are determined self-consistently. Such an approach is costly and in addition has the disadvantage that it does not conserve the orthogonality between wavefunctions of different states. The latter problem can be avoided by doing a PTED calculation for one particular state that is used as reference and determining the transition energies to other states by response theory. This approach also allows us to include the solvent-mediated screening of the coupling between different states via the linear response of the reaction field.^360^ The latter term depends on the transition densities and cannot easily be included in state-specific PTED-like calculations. The adaption of the reaction field to the changes in the solute’s charge density upon excitation can be accounted for by state-specific energy corrections. Such a combination of PTED with the corrected linear response (cLR) approach has been implemented in the ricc2 program for COSMO-RI-ADC(2) for excitation energies and one-photon spectra.^360^

Still, the macroiterations around the SCF and the post-SCF calculation (e.g., Hartree–Fock and CC2) make this approach costly. In particular, this hinders the calculation of gradients and other derivatives that would require tight thresholds for the self-consistency of the reaction field potential to be numerically stable. On the other hand, if the ground state is chosen as the reference state for PTED+cLR calculations, the SCF reaction field will be close to that from a fully self-consistent PTED calculation so that it is usually sufficient to include correlation effects on the reaction field only within the post-SCF correlation treatment. This post-SCF reaction field scheme was first proposed in Ref. 361 to combine RI-CC2 with the polarizable embedding (*vide infra*) and later^362,363^ also used with COSMO-CC2, and PE- and COSMO-ADC(2). It is not only cheaper than the PTED+cLR approach but can also readily be extended for the calculation of higher-order properties such as two-photon transition moments^364^ and derivatives, e.g., nuclear gradients,^362,363^ for ground and excited-state geometry optimizations.

A precondition for the applicability of any SCRF method, no matter if the environment is described by a continuum solvation model or atomistic embeddings, is that the excitation is localized on that part of the system that is quantum mechanically described. For continuum solvation models, this excludes Rydberg states. For the possibilities and limitations to describe Rydberg states with polarizable embedding models, we refer to Ref. 365.

### U. Electronic spectroscopy in solution: COSMO-ADC(2) and COSMO-CC2

In addition to the possibility to compute vertical excitation energies and transition moments, the implementation of COSMO-ADC(2) within the post-SCF scheme provides the ability to investigate the properties related to potential energy surfaces such as excited-state equilibrium geometries and de-excitation pathways of molecules in solution.^362^ In the pure linear response treatment, i.e., excluding the state-specific correction of the so-called corrected linear response approach, this approach produces physically correct potential energy surfaces in the vicinity of (near) degeneracies and conical intersections between excited states.^362^ With the post-SCF reaction field scheme, the additional computational cost for taking solvent effects into account at the COSMO-ADC(2) level on excitation energies and excited-state analytic gradients is a small portion of the corresponding gas phase calculations.

Another new approach in TURBOMOLE for including the solvent effects on spectra is COSMO-CC2 within the post-SCF reaction field scheme. It can be used as an alternative to COSMO-ADC(2) to compute vertical excitation energies and transition moments in solution within the framework of coupled-cluster response theory. With this approach, one can also calculate two-photon and magnetic circular dichroism (MCD) spectra of molecular systems in solution.^274^

**FIG. 30.**
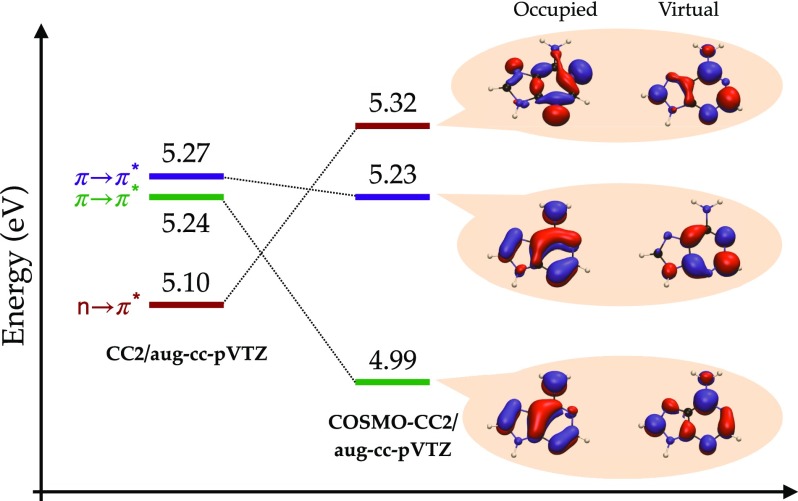
Vertical excitation energies of adenine in vacuum and aqueous solution at CC2/aug-cc-pVTZ using the COSMO solvent model within the post-SCF scheme along with the corresponding natural transition orbitals. Reprinted (adapted) with permission from Khani *et al.*, J. Chem. Theory Comput. **15**, 1242–1254 (2019). Copyright 2019 American Chemical Society.

Figure 30 highlights the application of COSMO-CC2 and CC2 on the spectrum of adenine.^274^ In vacuum at the CC2/aug-cc-pVTZ level, the lowest excitation, characterized as n → π^*^, is predicted at 5.10 eV, followed by two almost degenerate π → π^*^ states at 5.24 eV and 5.27 eV. The inclusion of the electrostatic interaction with an isotropic polarizable environment by the COSMO solvent model blue-shifts the n → π^*^ state to 5.32 eV, while the two π → π^*^ transitions are red-shifted to 4.99 eV and 5.23 eV respectively.

### V. Polarizable embedding

In addition to PE-CC2, the post-SCF reaction field scheme has also been employed in the implementation of polarizable embedded ADC(2) excited-state gradients.^363^ A comparison with supermolecular full-quantum mechanics (QM) calculations indicates that the ground and excited state geometry optimizations, at the polarizable hybrid quantum mechanics/molecular mechanics (QM/MM) PE-MP2 and PE-ADC(2) levels, respectively, are accurate enough for qualitative photophysics and photochemistry studies in complex molecular environments if proper parameterized electrostatic potentials, distributed polarizabilities, and van der Waals parameters can be provided.^363^ Moreover, PE-ADC(2) and PE-CC2 can be used for the calculation of transition moments and UV/vis spectra in atomistic solvent and biomolecular environments with the post-SCF reaction field scheme. The two-photon cross sections have also been implemented at the post-SCF PE-CC2 level. PE-ADC(2) and PE-CC2 methods are implemented at the quasirelativistic two-component level,^111,112^ albeit with a coupled-cluster Lagrangian that has been simplified for the purpose of polarizable embedding (sPE).^366^ This Lagrangian has the advantage that it is linear in the multipliers instead of quadratic. It can also be used in non-relativistic and scalar-relativistic one-component calculations.

**FIG. 31.**
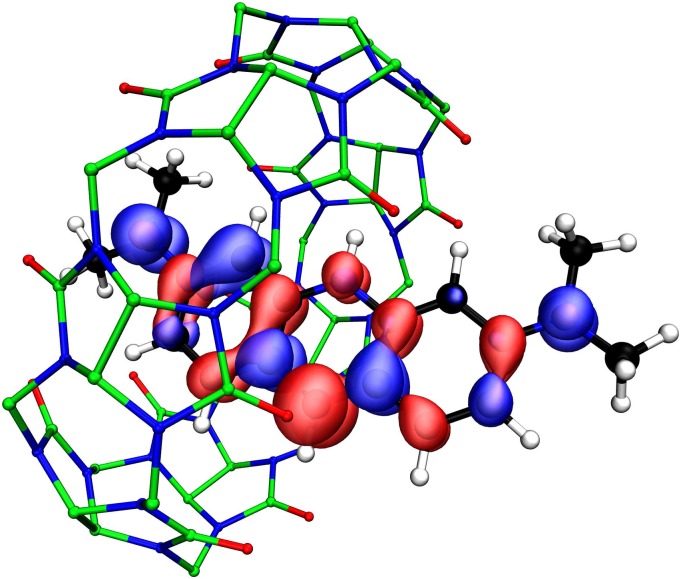
QM/MM excited-state geometry optimization has been performed at the PE-ADC(2)/TZVP level. The occupied and virtual natural transition orbitals are represented by blue and red with 0.05 a.u. isosurface value, respectively.

The electrostatic potential of the MM subsystem can be described by a given set of point charges, dipoles, and quadrupoles. The *ab initio* distributed multipoles can be calculated based on the intrinsic atomic orbital localization with the proper program, and the isotropic dipole–dipole polarizabilities can be taken from the D3 parameterization,^152^ which are readily available and do not need further calculations. The program can accept both isotropic and anisotropic dipole–dipole polarizabilities. Furthermore, in addition to the standard input format, TURBOMOLE is able to read the DALTON^367,368^ input for the polarizable embedding potentials, and thus, it is possible to perform PE calculations with *ab initio* potentials that are calculated based on molecular fractionation with the conjugated caps (MFCC) procedure for extended systems e.g., proteins, DNA, etc., using the PyFraMe^369^ utility. In addition, the van der Waals interactions between the QM and MM subsystems can be included in the input of the program. For instance, the fluorescent emission energy of gaseous acridine orange (AOH+) encapsulated in the macrocyclic cucurbituril[7] (CB7) molecular container, calculated with PE-ADC(2)/TZVP with the IAO-D3^152,370^ (calculated at the B3LYP/aug-ccpVDZ level) potential and OPLS Lennard-Jones parameters,^371,372^ is 2.47 eV. The experimentally measured value in the gas phase is 2.53 eV.^373^ Figure 31 shows the minimum of the lowest electronically excited potential energy surface of the AOH + @CB7 complex.

A common issue in QM/MM calculations that hinders accuracy is that electrons spill out of the boundaries of the QM and MM subsystems, which occurs due to the lack of a description for Pauli repulsion at the MM atoms. To improve the robustness and accuracy of QM/MM calculations of excitation energies, the atoms of the MM subsystem can be augmented with atom-specific ECPs that are parameterized for QM/MM calculations, which represent all electrons of the MM subsystem and do not require further parameterization. A benchmark study shows that the electron spill-out issue can be diminished even for the QM/MM calculation of Rydberg states in solution with diffuse basis sets.^365^

## IV. INTERFACES AND SOFTWARE ECOSYSTEM

### A. Generic import from and export to other software

Despite the fact that many quantum chemistry programs use Gaussian basis sets, raw data used internally such as the molecular orbitals, density, and electrostatic and other properties are stored in different formats and partly using different normalization schemes. Thus, various interfaces and post-processing scrips are provided. TURBOMOLE’s coordinate file can be converted to a standard Cartesian coordinate format (xyz) in ångström or to the input for Molden^374,375^ by internal scripts. xyz files can be used for plotting the molecular structure by Avogadro,^376,377^ Jmol,^378^ and VMD,^379^ to name only a few. Further conversion to pdb, cml, sdf, cif, the cube format (cub) or for the use of Open Babel,^380,381^ and NOMAD^382,383^ is available. The default format for plotting (localized) molecular orbitals or spinors, natural transition orbitals, (spin) densities, and the electrostatic potential or field on a grid is plt, which can be used by gOpenMol^384,385^ and VMD. To allow for the use of other external programs such as ParaView,^386^ these quantities can be written to disk as a plv, plx, map, dtx, txt, or cub file or converted to input files for AOMix,^387,388^ sTDA,^389,390^ and wfn files for AIMAII^391^ to analyze molecular orbital data. An interface to gCP^392,393^ for geometrical counterpoise corrections is provided. Moreover, post-processing scripts to study excited states are provided for ezSpectrum^394^ and TheoDORE;^395^ all of these post-processing scripts are available at the TURBOMOLE website.^43^

### B. Graphical user interfaces

**FIG. 32.**
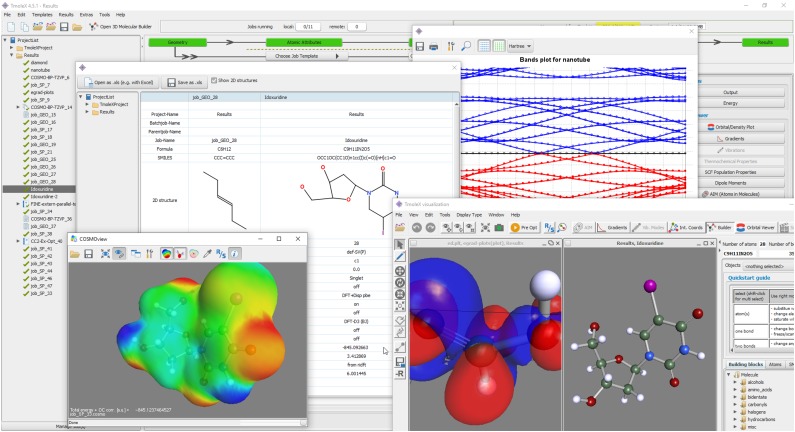
Graphical user interface TmoleX version 4.5.1 (2019).

Besides TURBOMOLE’s command line interface, which includes interactive input generation module define as the oldest but most flexible option or the more automated calculate script, the user-friendly Java and OpenGL based graphical user interface TmoleX^396^ is available for GNU/Linux, Windows, and MAC operating systems. TmoleX has been developed by COSMOlogic, and the client version is freely available for download on the web.^397^ A TmoleX screenshot is displayed in Fig. 32. TmoleX is intended for both beginners and advanced practitioners, trying on the one side not to hide the complexity of quantum chemistry and to provide a plethora of available options in TURBOMOLE but to ease the usage and to create self-defined templates and workflows for daily use on the other side. An important feature of TmoleX is the possibility to start single- and multi-jobs either locally using an internal queuing system or on remote servers or clusters with or without queuing systems such as Grid Engine, SLURM, LSF, or PBS.

The TmoleX workflow starts with the definition of the input geometry by importing coordinate files in various formats or by entering SMILES or by drawing or sketching the structures using the provided 3D and/or 2D builders, including the possibility to generate and visualize periodic boundary conditions. Preoptimization of molecules based on the universal force field (UFF),^398^ MOPAC,^399^ or xtb-GFN2,^400^ and the choice of most job types such as geometry optimizations, generation of spectra of various kinds, search for transition states, constraint optimizations, and scans along one or several internal coordinates as well as generation and visualization of orbitals, densities, natural transition orbitals, electric fields and other vector fields, population properties, and other post-processing tools such as Atoms-in-Molecules (AIM) are available.^401^

Thus, TmoleX is able carry out all steps associated with a calculation including the visualization and analysis of the results. Templates are available to perform routine calculations, which can also be applied to a batch of geometries to perform the same kind of calculation(s) on many molecules at a time. Various export formats and interfaces are provided.

The proprietary MAPS QUANTUM toolkit^402^ also offers a dedicated graphical user interface for TURBOMOLE.

### C. Ring currents: TURBOMOLE meets GIMIC

Magnetic fields force the electrons to move inside molecules, which leads to a magnetically induced current density. The knowledge of the resulting current pathways helps us to better understand their magnetic and electronic properties. Such calculations can be done with the gauge-including magnetically induced currents (GIMIC) method,^403–406^ which is interfaced to several electronic structure programs such as TURBOMOLE. GIMIC uses the basis set information, molecular structure, the unperturbed ground-state density, and the magnetically perturbed density as input, with the latter being obtained in chemical shielding calculations.

The analysis of the current density requires multiple steps as visual inspections of the current flow, e.g., in a plane above the molecule, can provide only qualitative information about the current pathways. The visualization depends on the distance of the plane above the molecule. Therefore, numerical integration of the current density can be used to determine the current strengths, which is a useful measure to quantify the amount of electron delocalization in the system and to characterize its aromaticity—even for multicyclic systems, where local and global ring currents are possible.^407^

**FIG. 33.**
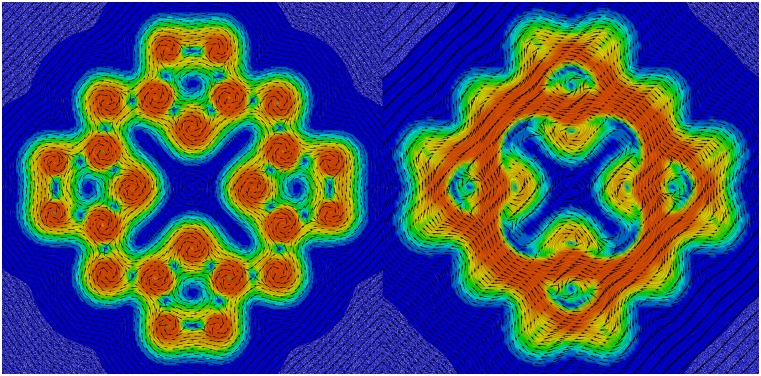
Ring currents in ${\left[{Hg}_{8}{Te}_{16}\right]}^{8-}$ (left) and in porphine (right), 1 bohr above the molecular plane, drawn between 0.00 a.u. (blue) and 0.07 a.u. (red). Reprinted with permission from Donsbach *et al.*, Angew. Chem., Int. Ed. **57**, 8770–8774 (2018). Copyright 2018 John Wiley and Sons.

The implementation of COSMO^26^ and ECPs^222^ (or X2C^95^) in TURBOMOLE’s NMR module made it possible to investigate the ring currents in the highly charged inorganic anion ${\left[{Hg}_{8}{Te}_{16}\right]}^{8-}$ to compare its electronic similarities and dissimilarities to porphine.^408^ Figure 33 shows the ring currents of the two systems in a plane 1 bohr above the molecules. The ${\left[{Hg}_{8}{Te}_{16}\right]}^{8-}$ anion exhibits weak local ring currents in the five-membered rings, arising from *σ*-contributions only, with a current strength of +5.8 nA/T. This is about half the strength of the ring currents in the benzene molecule. The global ring current strength around the entire molecule is close to zero. In contrast, porphine on the right side of Fig. 33 has a global ring current strength of about +27 nA/T.

Magnetically induced current densities for large toroidal carbon nanotubes with up to 2000 carbon atoms have been calculated using GIMIC and TURBOMOLE to investigate the influence of structural parameters on the ring current strengths.^409^ With the recent improvements in the NMR module,^26^ the numerical evaluation of the current density with GIMIC is now by far the time-determining step.

### D. Interfaces to QM/MM drivers

There are several QM/MM drivers that have interfaces to TURBOMOLE as quantum chemistry engine. For example, the Chemistry at HARvard Macromolecular Mechanics (CHARMM) force field^410^ has a dedicated TURBOMOLE interface;^411^ others include the GROningen MAchine for Chemical Simulation (GROMACS),^412–418^ ChemShell,^419,420^ Atomic Simulation Environment (ASE),^421^ and NAMD.^422,423^ The TURBOMOLE trajectories can be analyzed and visualized with the TRajectory Analyzer and VISualizer (TRAVIS).^424^ We refer the interested readers to the corresponding references for further information about the details of the QM/MM coupling schemes, the supported functionalities, and license conditions for the QM/MM drivers and/or MM codes.

### E. Molecular dynamics simulations

TURBOMOLE comes with frog, a dedicated module for *ab initio* molecular dynamics (AIMD).^425,426^ frog uses the leapfrog-Verlet integrator and supports microcanonical and canonical (Nosé–Hoover) Born–Oppenheimer molecular dynamics, constrained dynamics, as well as simulated annealing, and sudden quenching for global minimum search. frog can be used for BO molecular dynamics simulations in conjunction with all electronic structure methods available in TURBOMOLE, providing analytical gradients. frog also supports multi-state nonadiabatic molecular dynamics using Tully’s FSSH,^330,345,427,428^ including anisotropic velocity re-scaling.

SHARC stands for “Surface Hopping including Arbitrary Couplings,” is developed and distributed by González and co-workers,^429–431^ and has been interfaced to the ricc2 code to run surface hopping *ab initio* molecular dynamics (AIMD) at the CC2 and ADC(2) level using isotropic velocity re-scaling. It also supports QM/MM calculations at these levels. The AIMD code NEWTON-X by Barbatti and co-workers^432,433^ also has a TURBOMOLE interface to perform nonadiabatic dynamics with the available methods.

## V. TURBOMOLE LICENSING AND DISTRIBUTION

### A. End-user licenses

TURBOMOLE GmbH holds the right to use, distribute, and commercialize the TURBOMOLE program suite and owns a significant fraction of the source code. TURBOMOLE GmbH was founded in 2007 and is governed by its currently five stakeholders who are also core developers of different parts of the code. TURBOMOLE GmbH has adopted an irrevocable bylaw preventing the distribution of dividends. This ensures that all profits are re-invested into the TURBOMOLE project and prevents the current and future generations of owners from enriching themselves at its expense. TURBOMOLE’s financial balance sheets are published yearly in Germany’s Commercial Register.^434^

End-user licenses offered for TURBOMOLE executables differ by the type of use (for example, educational, academic, non-profit, and for-profit) and the number or scope of users. TURBOMOLE licenses are either perpetual or time limited, and licenses for education and training are free of charge. New TURBOMOLE releases are published approximately once yearly. New features and release notes are available at the TURBOMOLE website.^43^

An important motivation for TURBOMOLE’s current business model was that individual user support by professionally trained staff is frequently requested by TURBOMOLE users. This not only includes help with installing and running TURBOMOLE but also consultation on specific applications users would like to perform. TURBOMOLE GmbH is currently under contract with COSMOlogic (now Dassault Systèmes) as its exclusive provider of user support and maintenance. Licenses with support are distributed by Dassault Systèmes. We refer to the website^43^ for details.

Apart from the costs of user support provided by Dassault Systèmes, TURBOMOLE license fees are meant to ensure long-term stability of the code by continued maintenance and development. Just as large multi-investigator international experimental scientific facilities finance their operation and upkeep through fees, the continuous license income is critical for the long-term stability and sustainability of the TURBOMOLE project.

### B. Source code license

TURBOMOLE GmbH distributes source code licenses free of charge for the sake of improving the program. The main requirement for obtaining a TURBOMOLE source code license is a brief proposal describing why the source code is sought. Typically, these proposals outline a specific code development project, but source code licenses can also be granted for other reasons, e.g., debugging, maintenance, or validation. TURBOMOLE is open for new developers, and anyone can apply directly to TURBOMOLE’s scientific coordinator(s).

The TURBOMOLE source code license is a simple reciprocal agreement: In exchange for access to the source code and the right to use it for research and teaching, the licensee grants TURBOMOLE GmbH the non-exclusive right to commercialize any additions they make to the source code and agrees to respect the rights of other developers and keep the source code confidential. TURBOMOLE does not expect developers to give up their ownership or rights to use or commercialize the code they have authored.

Proposals for source code licenses are reviewed by TURBOMOLE GmbH. Important review criteria include (i) scientific soundness and merit, (ii) alignment with goals of the TURBOMOLE project, (iii) no overlap or potential conflicts with on-going approved code development projects, and (iv) demonstrated willingness to contribute to the TURBOMOLE project. Development proposals can also receive limited financial support from TURBOMOLE if they make a compelling case.

Compared to classical “open source” licenses, TURBOMOLE’s source code and end user licenses are more restrictive in that they do not allow free and anonymous download from a web repository. The goals of this licensing policy are (i) enabling user support and maintenance, (ii) ensuring long-term stability of the code, (iii) fostering collaboration and trust among developers, and (iv) providing adequate access to the source code and/or executables for research and teaching. For a detailed discussion of licensing models, the reader is referred to Ref. 435.

## VI. TURBOMOLE DEVELOPMENT

TURBOMOLE developers recognized the importance of version control for shared code development and reproducibility early on: TURBOMOLE development has been version-controlled since 1992, its version history comprising more than 20 000 unique commits. TURBOMOLE version control has been based on Git^436,437^ and GitLab^438^ since 2018. A second important element of TURBOMOLE development is its automated test suite, which will run hundreds of tests and compare them to reference output to help validate a version. Since 2019, TURBOMOLE is built and the test suite is deployed automatically after each merge into the master branch.

TURBOMOLE follows a simple development workflow for adding a new feature:

1. Inform the developers community and start a feature branch.
2. Implement the new feature locally.
3. Add documentation.
4. Ensure the feature branch passes the test suite on at least two different platforms.
5. Open merge request and assign to a reviewer.
6. Merge feature branch into master after positive review.

Similar workflows exist for bug fixes or creating a new release version.

The majority of TURBOMOLE source code is written in Fortran 90, although features of newer Fortran releases are increasingly adopted. Fortran continues to be used due to its simple syntax, backward compatibility, and highly optimized compilers for high-performance computing. Additionally, Fortran is close enough to the assembler to allow for targeted optimization of time-critical code such as integral routines.

## VII. CHALLENGES

The TURBOMOLE project has been remarkably resilient, overcoming vast changes in hardware, software, and its developer and user communities during the past three decades. However, with the size of its code, user, and developer bases, TURBOMOLE’s problems have grown as well.

### A. Communication and outreach

In the past 15 years, TURBOMOLE has transitioned from a code developed by a single principal investigator and a few collaborators to an organization spanning three continents and over 50 active developers. This increase in geographic and scientific diversity comes at the cost of communication. Establishing a culture of communication and collaboration among developers across different countries and backgrounds has been challenging because it requires extra time and effort. In an attempt to address this challenge, TURBOMOLE GmbH has organized developers’ meetings and supported the exchange of students among groups of developers.

To help build trust and community among its developers, TURBOMOLE requires confidentiality as a condition for access to its source code, providing a measure of protection and encouraging early internal sharing of new code. Moreover, an important evaluation criterion for new development is that any proposed project must not compete with existing, already approved development proposals in good standing. While these agreements have been useful to avoid a cut-throat “wild west” environment, they are sometimes interpreted differently among developers. In practice, it still happens too often that original authors are not adequately credited for their work, which causes reservations about sharing new developments quickly and widely.

Outreach has been a relative weakness of TURBOMOLE from its beginnings. To some extent, this can be traced to TURBOMOLE’s culture of proud understatement and the notion of being an underdog or “mole” challenging other, perhaps more established software suites. Moreover, many TURBOMOLE developers primarily see themselves as scientists who would rather spend time on improving the code than marketing it to the world. Especially in the early years, this attitude has helped motivate TURBOMOLE developers and establish it as a tool of choice for experts performing breakthrough applications; however, as the code has become more mature, the same attitude has impeded dissemination of TURBOMOLE to a broader community of uninitiated users.

Successful outreach depends, to an extent, on the willingness of the primary developers to write user-friendly code, and document, explain, and illustrate the use of their code. While TURBOMOLE GmbH can provide incentives to its developers, documentation and outreach are likely to remain challenging until the importance of robust and well-documented software infrastructure is more broadly recognized by the scientific community. This includes users and funding agencies who understand that professional software and user-friendly interfaces take effort to develop and maintain and does not come for free. Similarly, professional users need to be willing to read instructions and educate themselves beyond a quick web search, resisting the narrative that merely purchasing the right software will solve all their problems.

TURBOMOLE developers have been predominantly European males. However, TURBOMOLE GmbH was founded to carry on the TURBOMOLE project “on many shoulders,” and the current generation of TURBOMOLE developers is more diverse than any before it. TURBOMOLE GmbH welcomes the participation of developers from non-traditional and minority backgrounds.

### B. Software infrastructure

Parts of TURBOMOLE code base are “legacy software” dating back to the 1980s. Constant bug-fixing as well as minor and occasional major re-writing of the existing code is necessary just to maintain the suite and adapt it to an ever-changing software and hardware environment. The need to maintain and improve code infrastructure often conflicts with the desire of individual researchers to have “their method” implemented with as little effort as possible.

It is far more difficult to separate “maintenance” from original research than one might assume based on commercial software development practice. This is a critical yet often overlooked difference distinguishing scientific software development at the cutting edge of research from more mundane application software development. While certain well-defined and isolated tasks can be outsourced to less skilled programmers, many supposedly minor changes involve design decisions requiring an intimate, high-level understanding of how and to what ends electronic structure software is used and where its limitations are. Moreover, as methods and theories evolve, they frequently require changes in algorithms. For example, as opposed to the present, it was inconceivable in the 1980s that basis sets containing *h* (*l* = 5) functions would be routinely used,^439,440^ and hard upper limits for *l*-quantum numbers were standard. Due to such “technical complications,” it often takes newcomers years to become highly effective at code development, whereas senior developers find themselves overwhelmed with maintenance requests because their expertise is at high demand.

Clear design principles, a modular program structure with encapsulated functionality, detailed documentation, and a supportive community of developers can all help to address this challenge. Just as important, however, is the recognition that a strict separation between efficient code development and fundamental, innovative science is impossible. Unlike in experimental science, where the need for maintenance can be somewhat obvious from the sheer size and complexity of scientific apparatus, it can be far less obvious to non-experts whether they are dealing with a well-maintained, reliable, and resource-efficient code or polished but inefficient, and unstable software.

### C. Funding

For high-quality collaborative software projects such as TURBOMOLE to be successful, they require funds to support code maintenance, to keep pace with evolving hardware architectures, and funds to support code development, to incorporate and disseminate new and useful scientific ideas and methods. TURBOMOLE GmbH uses the portion of the revenue from license fees left over after costs of distribution and user support are deducted to fund code development projects, but this has typically been a budget of under EUR 300,000 per year,^434^ which is comparatively small considering the size of the code and the number of developers.

At the time of writing this article, the TURBOMOLE project is supported to a large extent by volunteer contributions from the developer community and through PI’s research grants that use TURBOMOLE as the platform in which new scientific ideas are implemented, tested, and applied. For better or worse, TURBOMOLE GmbH has never been the recipient of dedicated grants for code development. TURBOMOLE GmbH is structured to keep administrative costs at an absolute minimum: it is run by volunteers with minimal or no compensation and tight time constraints, even the CEOs are part-time, and there is no dedicated office suite, and this often means that only the most urgent organizational tasks get done. The importance of a consistent income stream for long-term maintenance and code stability is hard to overstate, and securing funding is an ongoing challenge.

The unorthodox use of a commercial entity, TURBOMOLE GmbH, as the vehicle for sustaining stability and longevity of the TURBOMOLE project oftentimes leads to a misunderstanding of TURBOMOLE’s aims. TURBOMOLE developers face the challenge of explaining to funding bodies and reviewers that the TURBOMOLE project is by the community and for the community and not a profit making enterprise and that development of new scientific ideas must coincide with the incorporation of those ideas into professional software so that they can be used effectively by current and future generations of scientists.

TURBOMOLE’s most important source of support is the enthusiasm of its developers, who continue volunteering their time to serve the scientific community and advance the TURBOMOLE project.

## SUPPLEMENTARY MATERIAL

See supplementary material for history of the project, overview of basis sets developed in conjunction with TURBOMOLE, and EPR capabilities.

## DATA AVAILABILITY
